# GM-CSF Production Allows the Identification of Immunoprevalent Antigens Recognized by Human CD4+ T Cells Following Smallpox Vaccination

**DOI:** 10.1371/journal.pone.0024091

**Published:** 2011-09-09

**Authors:** Valeria Judkowski, Alcinette Bunying, Feng Ge, Jon R. Appel, Kingyee Law, Atima Sharma, Claudia Raja- Gabaglia, Patricia Norori, Radleigh G. Santos, Marc A. Giulianotti, Mark K. Slifka, Daniel C. Douek, Barney S. Graham, Clemencia Pinilla

**Affiliations:** 1 Torrey Pines Institute for Molecular Studies, San Diego, California, United States of America; 2 Torrey Pines Institute for Molecular Studies, Port St. Lucie, Florida, United States of America; 3 Vaccine and Gene Therapy Institute, Oregon Health and Science University, Beaverton, Oregon, United States of America; 4 Vaccine Research Center, National Institute of Allergy and Infectious Diseases (NIAID), National Institutes of Health (NIH), Bethesda, Maryland, United States of America; Centre de Recherche Public de la Santé (CRP-Santé), Luxembourg

## Abstract

The threat of bioterrorism with smallpox and the broad use of vaccinia vectors for other vaccines have led to the resurgence in the study of vaccinia immunological memory. The importance of the role of CD4+ T cells in the control of vaccinia infection is well known. However, more CD8+ than CD4+ T cell epitopes recognized by human subjects immunized with vaccinia virus have been reported. This could be, in part, due to the fact that most of the studies that have identified human CD4+ specific protein-derived fragments or peptides have used IFN-γ production to evaluate vaccinia specific T cell responses. Based on these findings, we reasoned that analyzing a large panel of cytokines would permit us to generate a more complete analysis of the CD4 T cell responses. The results presented provide clear evidence that TNF-α is an excellent readout of vaccinia specificity and that other cytokines such as GM-CSF can be used to evaluate the reactivity of CD4+ T cells in response to vaccinia antigens. Furthermore, using these cytokines as readout of vaccinia specificity, we present the identification of novel peptides from immunoprevalent vaccinia proteins recognized by CD4+ T cells derived from smallpox vaccinated human subjects. In conclusion, we describe a “T cell–driven” methodology that can be implemented to determine the specificity of the T cell response upon vaccination or infection. Together, the single pathogen *in vitro* stimulation, the selection of CD4+ T cells specific to the pathogen by limiting dilution, the evaluation of pathogen specificity by detecting multiple cytokines, and the screening of the clones with synthetic combinatorial libraries, constitutes a novel and valuable approach for the elucidation of human CD4+ T cell specificity in response to large pathogens.

## Introduction

Vaccinia virus (VACV) is the virus used in the human smallpox vaccine. Considered the gold standard of vaccines, it was very effective in bringing about the worldwide eradication of smallpox disease. This vaccine, (Dryvax), is one of the two FDA approved vaccines to smallpox in the United States (ACAM2000 was approved in 2007). However, its use is hampered by the risk of adverse effects and even some mortality [1]. Significant efforts have been focusing on both the understanding of the immune response to Dryvax/ACAM2000 and the evaluation of alternative vaccines such as modified vaccinia Ankara (MVA).

Humans make strong CD8+ and CD4+ T cell responses after receiving smallpox vaccination. The kinetic analysis suggests that CD4+ responses are lower than CD8+ responses at 2 weeks post-vaccination [2], but similar in magnitude at 1 month post-vaccination [2]–[4]. Furthermore, both vaccinia specific neutralizing antibodies and CD4+ responses are detected in subjects after more than 40 years of Dryvax immunization or variola infection [4]–[7]. It is also well established that CD4+ T cells are important in primary clearance of vaccinia and in the induction and maintenance of long-term memory and protection from variola challenge. In addition, CD4+ T cells assist antigen specific antibody production, and antibodies are necessary for vaccine-induced protection to orthopoxvirus challenge. It has been recently demonstrated in mice that antibody specificity in response to vaccinia virus is determined by intramolecular protein-specific CD4+ T cell help [8]. In other words, CD4+ T cell responses to a given protein are required for the production of antibodies to the same protein.

The importance of the role of CD4+ T cells in the control of vaccinia infection is well known, however more CD8+ than CD4+ T cell epitopes recognized by human subjects immunized with vaccinia virus have been reported (search performed on January 5^th^, 2011 in the Immune Epitope Database website, (www.iedb.org)). This is probably due to the fact that prediction binding algorithms that have been largely used to identify CD8+ specific T cell antigens have relatively poor prognostic ability for predicting peptides that bind class II molecules [9], [10]. The vaccinia CD4+ T cell epitopes described up to now have been derived using: 1- candidate proteins and overlapping peptides [11], [12]; 2- recombinant antigens covering the entire predicted vaccinia virus proteome to screen CD4+ T cell clones from vaccinated donors [13]–[15]; 3- MHC Class II binding predictions for HLA-DR1 restricted peptides predicted by a combination of P9 and Syfpeithi binding algorithms [16], [17] or EpiMatrix algorithm [18]; and 4- a two-dimensional liquid chromatography and tandem mass spectrometry approach used to identify vaccinia virus-derived peptides among all the peptide antigens bound to the human class II MHC protein HLA-DR1 on the surface of vaccinia virus infected cells [19].

Most of the studies that have identified human CD4+ specific protein-derived fragments or peptides have used IFN-γ production by intracellular staining (ICCS) or ELISPOT to evaluate vaccinia specific T cell responses. However, a recent study analyzing immunological memory in a number of volunteers that have been variola infected or vaccinia immunized demonstrated that in the majority of the samples there was no correlation between the proliferation to vaccinia and the presence of IFN-γ-producing T cells [5]. Interestingly, Slifka and collaborators have observed clear intracellular TNF-α production in response to vaccinia infection by in vitro stimulated peripheral CD4+ T cells from vaccinated donors [4]. Based on these findings, we reasoned that analyzing a large panel of cytokines in response to vaccinia would permit a more complete study of T cell responses that could not have been detected if evaluating only IFN-γ production. In fact, the results presented here confirm that TNF-α is an excellent readout of vaccinia specificity and that other cytokines such as GM-CSF can be used to evaluate the reactivity of CD4+ T cells in response to vaccinia antigens. In this study we also present the elucidation of 4 novel vaccinia epitopes by directly assessing the specificity of CD4+ T cells derived from vaccinia immunized subjects without any assumption regarding antigen binding restriction and independently of previous identified immunogenic candidate proteins. This unbiased “T cell driven” approach used vaccinia specific CD4+ T cell clones generated from vaccinated donors and subsequently screened with a decapeptide positional scanning library. The screening results in conjunction with the biometrical analysis [20], [21] performed with a vaccinia virus Western Reserve protein database resulted in the identification of 4 novel highly conserved vaccinia human CD4+ T cell epitopes. Furthermore, a comprehensive analysis of the reported immunogenicity of the vaccinia proteins from which the identified epitopes are derived revealed that the “T cell driven” approach presented here results in the identification of peptides derived from highly immunogenic vaccinia proteins. This “T cell driven” approach can be readily implemented to determine the specificity of the response following vaccination or infection with large size pathogens for which other methodologies could be more cumbersome.

## Results

### Cytokine production by vaccinia specific T cell lines and CD4+ T cell clones in response to vaccinia infected antigen presenting cells

**Figure 1:**
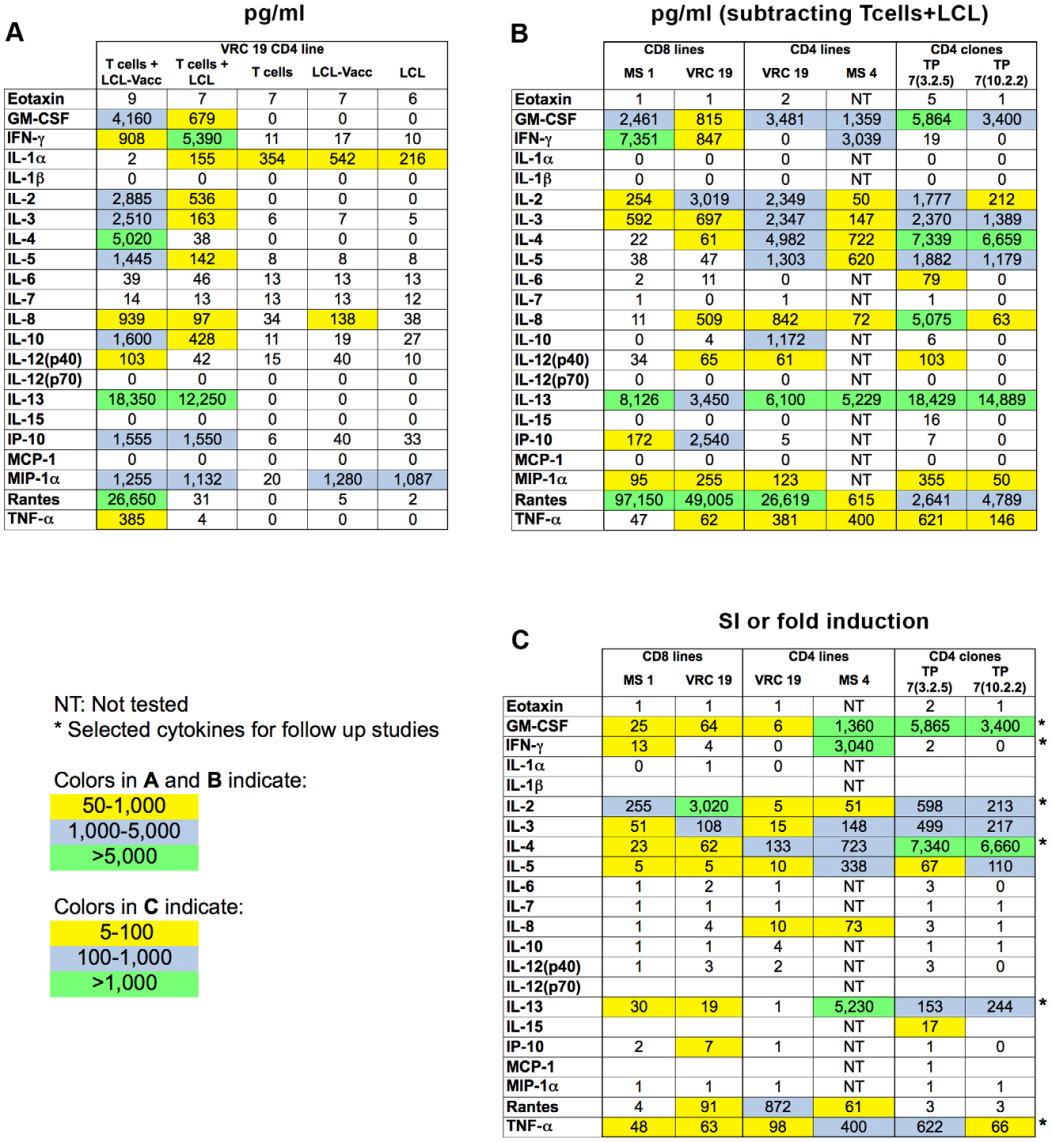
Cytokine production by CD4+ and CD8+ T cell lines and clones in response to antigen presenting cells infected with vaccinia. T cells lines and clones were cultured alone or in the presence of autologous LCL (background values) or vaccinia infected LCL. Supernatants were collected after 48 hours of stimulation and cytokine production was evaluated by multiplex assay as described in *Materials and Methods*. A. Cytokine production by VRC 19 CD4+ lines and control cultures is expressed in pg/ml. B. Normalized values resulting from the subtraction of background values from pg/ml produced by various lines and clones in response to vaccinia infected LCL are shown. C. Each value represents the stimulation index (SI) resulting from dividing the pg/ml produced in response to vaccinia infected LCL by the background values.

A reliable and representative readout was developed to test the vaccinia specific reactivity of T cell lines and clones derived from PBMC of vaccinia immunized subjects [22]. Cytokine production was chosen because it can be easily measured in a large number of culture supernatants. The production of 22 cytokines was simultaneously evaluated by a cytokine multiplex assay. Culture wells included vaccinia specific CD4+ or CD8+ T cell lines or CD4+ T cell clones derived from VRC19 donor in the presence of uninfected autologous LCL or vaccinia infected autologous LCL (LCL-Vacc). Figure 1A shows the concentration of each cytokine produced by a VRC19 CD4+ T cell line in response to LCL-Vacc or uninfected LCL. Additional controls included T cells, LCL-Vacc or uninfected LCL alone. Fourteen cytokines were produced in response to vaccinia infected LCL. Although at lower levels, cytokines were also detected when T cells were cultured with uninfected LCL. With the exception of IL-1α, IL-8 and MIP-1α, no cytokines were detected in the wells with T cells, LCL-Vacc or uninfected LCL alone. To determine the response to vaccinia by the VRC19 CD4+ line as well as other CD4+ and CD8+ lines and two T cell clones, the T cell response value to uninfected LCL was subtracted from the T cell + LCL-Vacc value (Figure 1B). Alternatively, the results were normalized by dividing the T cell+ LCL-Vacc value by the T cells + uninfected LCL background value to give a stimulation index (SI) for each cytokine (Figure 1C). As this study focused on CD4+ T cell specificity, only those cytokines that were produced by CD4+ T cell lines or clones were selected. As shown with the color formatting in Figure 1, GM-CSF, IL-2, IL-3, IL-4, IL-13 and TNF-α were detected in high levels by most CD4+ T cell lines and clones in response to vaccinia stimulation. IFN-γ was clearly detected in the supernatants of CD8+ T cell lines and by one CD4+ T cell line (MS4) but was not detectable in other CD4+ T cell clones. Based on these results and on reagent cost considerations, 6 cytokines, namely GM-CSF, IL-2, IL-4, IL-13, TNF-α and IFN-γ were selected to evaluate the vaccinia specificity of the CD4+ T cell clones. Even though the CD4+ T cell clones analyzed for the production of 22 cytokines did not show IFN-γ production, IFN-γ was included due to its known function in antiviral immunity [23] and its frequent use in the evaluation of specific T cell responses [24], including CD4+ responses to vaccinia [11], [13], [16], [17].

**Figure 2:**
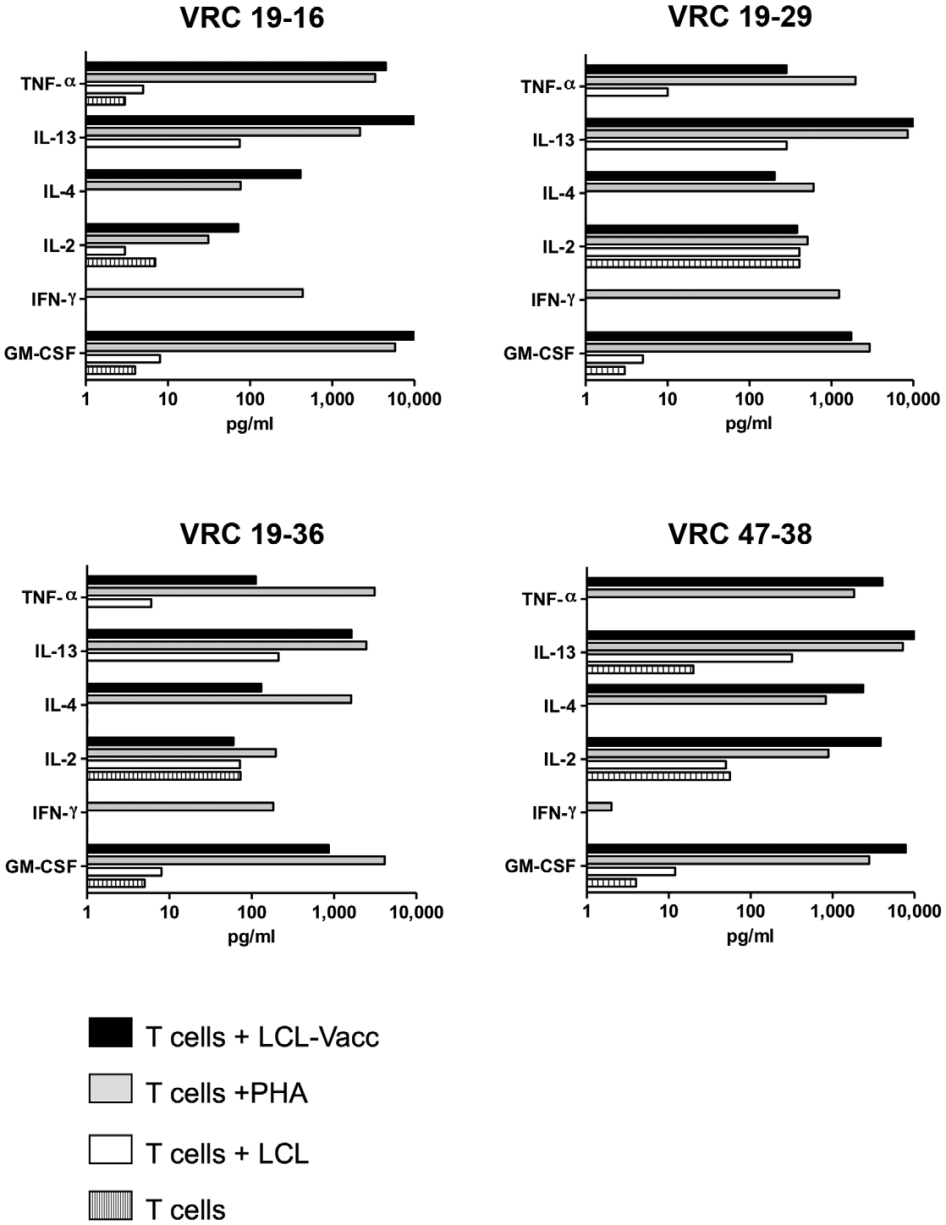
Cytokine production by vaccinia specific CD4+ T cell clones in response to vaccinia infected autologous LCL. Clonal T cells (2.5×10^4^) were cultured alone, stimulated with PHA or in the presence of 5×10^4^ autologous LCL or vaccinia infected LCL (LCL-Vacc). Supernatants were collected after 48 hours and cytokine production was evaluated by multiplex assay. Data represents the average of the cytokine concentrations determined in the supernatant of two replicate culture wells.

Vaccinia lines from 3 vaccinia immunized subjects were used to generate T cell clones, and 45 clones were established and expanded. The clones were selected based on vaccinia reactivity and growing characteristics. The elucidation of the specificity of 4 clones and the antigen specificity of a total of 9 clones is presented here. Clones VRC19-16, VRC19-29, VRC19-36 were derived from donor VRC19 and clone VRC47-38 from donor VRC47. As shown in Figure 2, the 4 clones showed clear cytokine production in response to autologous LCL-Vacc and PHA. However, consistent with the results presented in Figure 1, IFN-γ was not detected in response to vaccinia infected LCL, whereas TNF-α, IL-13 and GM-CSF were produced in a virus specific manner. GM-CSF and TNF-α were clearly produced in response to vaccinia. IL-4 was produced at lower levels by all 4 clones in response to vaccinia. IL-2 was produced by all the clones in response to vaccinia and PHA but at levels comparable to those of background values, except for clone VRC 47-38. Based on these results, GM-CSF and TNF-α production were selected to further evaluate the response of the clones to vaccinia infection and for the screening of the positional scanning library.

### Elucidation of vaccinia T cell clones antigen specificity by positional scanning peptide libraries

**Figure 3:**
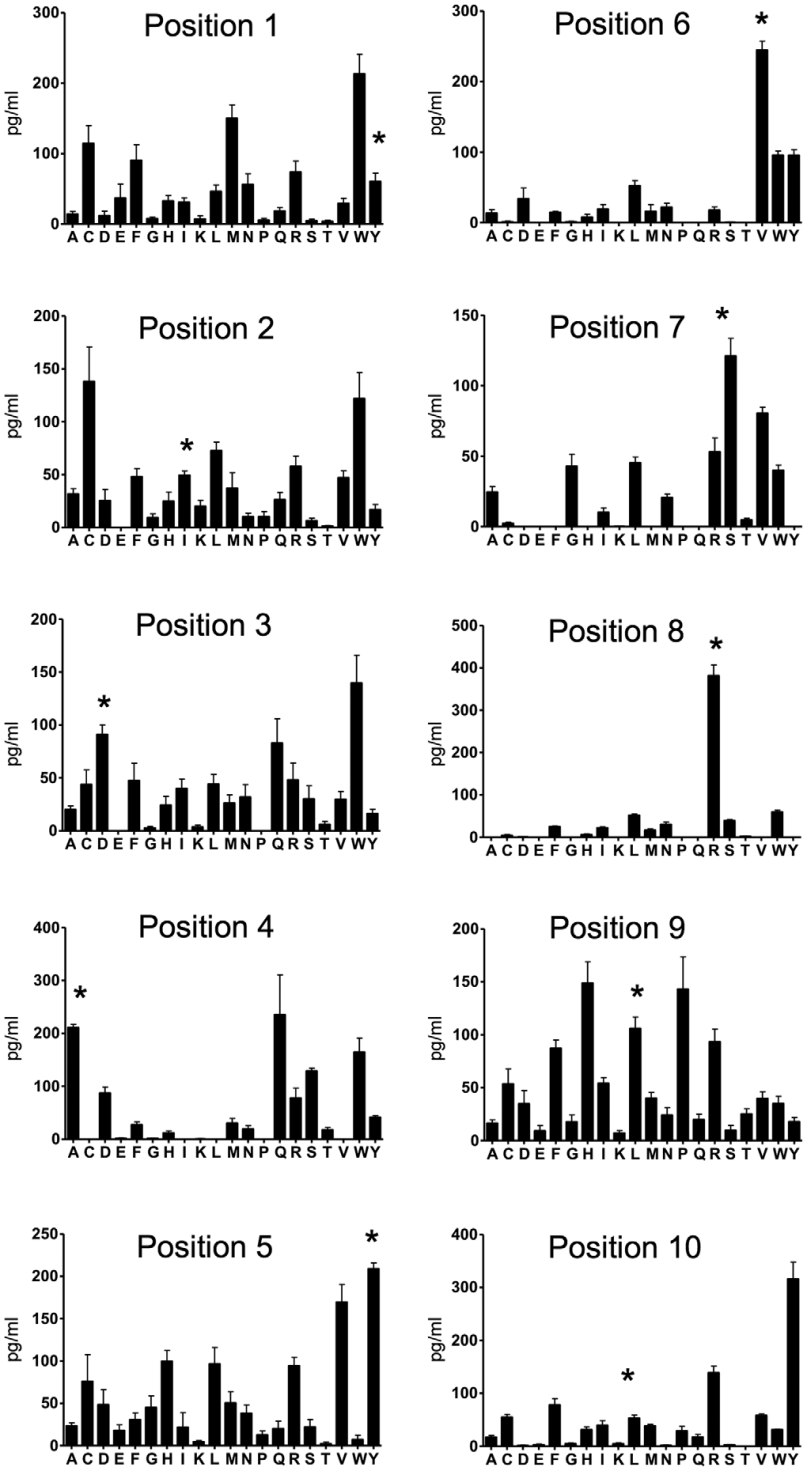
Screening profile of clone VRC19-16 in response to the decapeptide positional scanning library. The GM-CSF production (pg/ml) elicited by VRC19-16 T cells in response to each mixture of the decapeptide library was measured. 2.5×10^4^ VRC19-16 T cells were cultured in the presence of 5×10^4^ autologous LCL and each library mixture at a final concentration of 200 µg/ml. After 48 hours the GM-CSF production was evaluated in the culture supernatants by ELISA. Values represent average of 3 different screenings with duplicate culture wells for a total of 6 replicates. The x axis in each plot indicates the defined amino acid (one letter code) in each of the mixtures of the library. In each position, the amino acid corresponding to the amino acid present in the identified vaccinia peptide D13L-YID is indicated (*).

In order to elucidate the specificities of T cell clones VRC19-16, VRC19-29, VRC19-36 and VRC47-38, each clone was screened with a decapeptide positional scanning library using autologous LCL as antigen presenting cells. At least two different screenings were carried out for each clone. All clones showed reproducible responses to most of the mixtures of the library as measured by GM-CSF production. Significant and reproducible TNF-α production was not detected in response to the peptide mixtures of the library (data not shown). Figure 3 shows the average values obtained from 3 screenings of clone VRC19-16 with each of the mixtures of the decapeptide library. The number of mixtures that induce GM-CSF production (active mixtures, >50 pg/ml) varies at each position. For example, the only active mixture in position 8 was defined with arginine (R). In positions 1, 4 and 9 more than 2 mixtures induced GM-CSF production with values >100 pg/ml. The results obtained for each of the clones in response to the decapeptide positional scanning library were organized in a matrix format (*as described in the Material and Methods*) and are shown in Table S1. The screening results were used to predict and rank vaccinia stimulatory peptides by performing the biometrical analysis [20], [21] as described in the *Materials and Methods* section. The biometrical analysis was performed using a custom Western Reserve Vaccinia protein database which consisted of 216 proteins and a total of 55,892 decapeptides which were scored using the matrix for each of the clones. For each individual clone the highest scoring vaccinia peptides (15–35 peptides of a total of 55,892 scored peptides) were synthesized and tested for their stimulatory potencies at 10 µg/ml and 1 µg/ml with their respective T cell clone in the presence of autologous LCL. Positive control wells with T cells + LCL-Vacc and negative controls with T cells + uninfected LCL were also included in each experiment. Table S2 summarizes the GM-CSF and TNF-α secretion levels for each of the clones in response to the peptides synthesized and tested for each clone. All clones responded to the first or second ranked predicted peptide. The identified peptides for each of the clones and the information for the proteins from which these peptides are derived are summarized in Table 1.

**Table 1:** Characteristics of identified vaccinia CD4+ epitopes.

Clone^a^	Epitope	VACV-WR	Protein	Residues	% Homology^b^	Description^c^	Abbreviation
VRC19-16 (V_β_8)	YIDAYVSRLL	VACV-WR118	D13L	283-292	100	rifampicin target associates with inner surface immature virus membrane	D13L-YID
VRC19-29 (V_β_4)	MYTYFSNTIL	VACV-WR057	E1L	396-405	100	poly-A polymerase catalytic subunit VP55	E1L-MYT
VRC19-36 (V_β_8)	SFWFLKSGAV	VACV-WR125	A6L	75-84	100	unknown	A6L-SFW
VRC47-38 (V_β_2)	DWVSSHSKSL	VACV-WR052	F13L	361-370	100	palmytilated EEV membrane protein; phospholipase motif, required for IEV formation	F13L-DWV

a V_β_ determination by FACS with exception of VRC19-16 determined by PCR.

b within vaccinia and variola strains.

c from Ortholog list in poxvirus.org.

**Figure 4:**
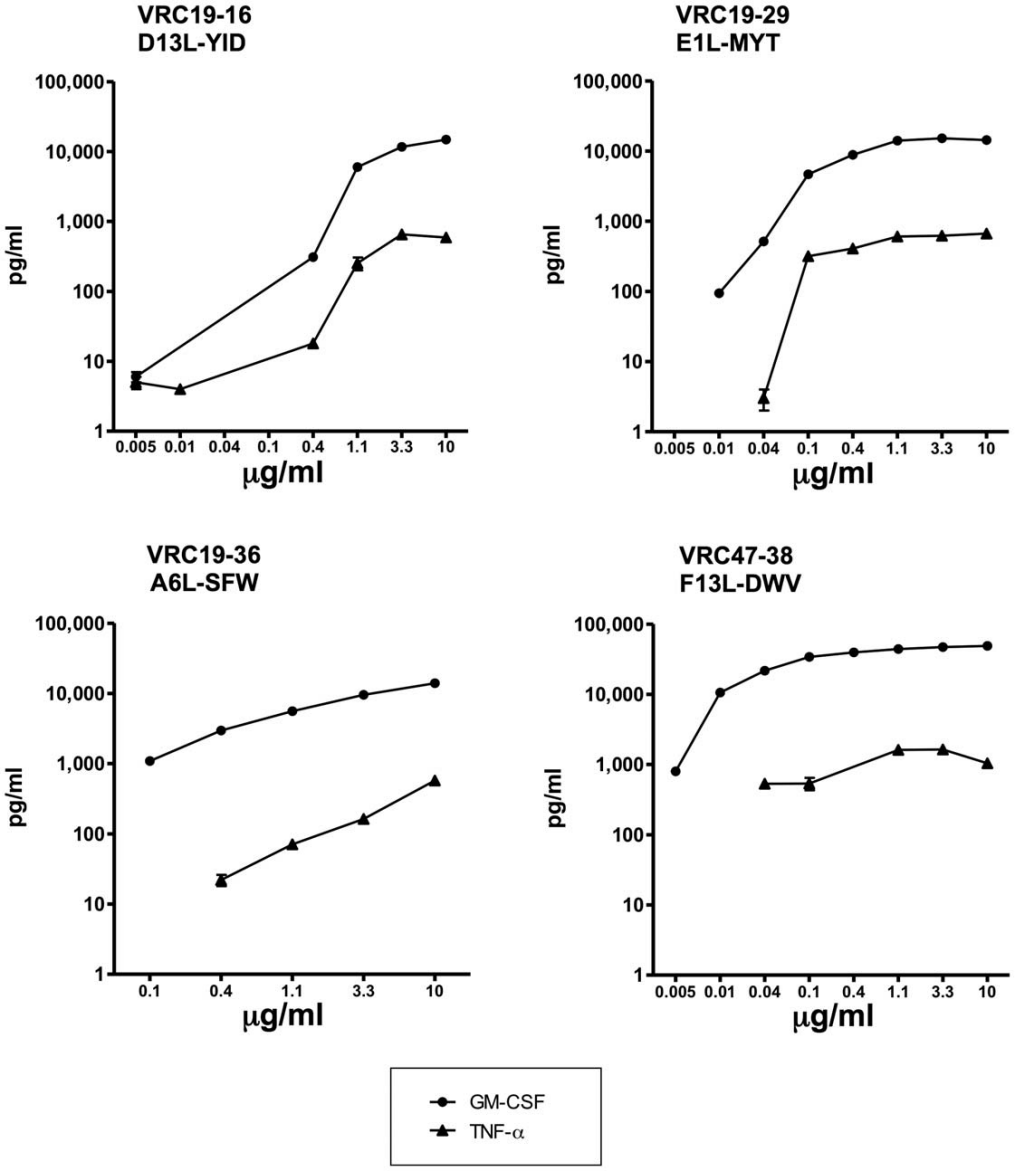
GM-CSF and TNF-α production by vaccinia specific CD4+ T cell clones in response to the identified vaccinia peptides. Each vaccinia CD4+ T cell clone (2.5×10^4^) was stimulated for 48 hours in the presence of autologous LCL (5×10^4^) and peptide in a dose response assay at the indicated concentrations. GM-CSF and TNF-α was measured in the culture supernatants by ELISA. Values represent the average cytokine production in two independent duplicate wells and are derived from one representative experiment out of three.

The stimulatory activity of the identified peptide for each clone was tested in a dose response manner with its respective clone. Figure 4 shows that the GM-CSF and TNF-α production are dose dependent for all the clones in response to peptide, and the peptides are highly stimulatory at low concentrations. Interestingly, for all the clones, GM-CSF production is detected at lower peptide concentrations than TNF-α production, which correlates with the observation that GM-CSF and not TNF-α was detected in response to peptide mixtures of the positional scanning library. In summary, 4 novel vaccinia CD4+ T cell epitopes have been identified using GM-CSF production by CD4+ T cells as the readout of activity to peptide mixtures of a decapeptide positional scanning library.

### Multi-cytokine production analysis in response to vaccinia CD4+ epitopes

**Figure 5:**
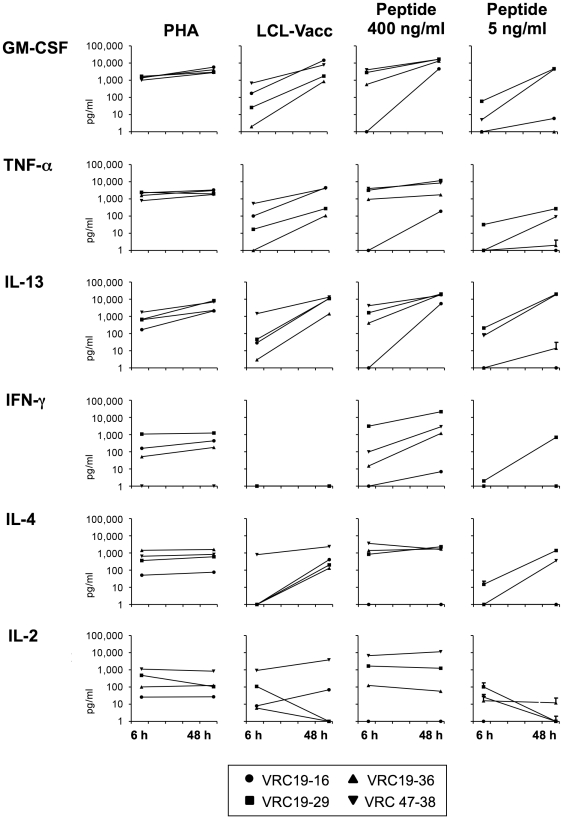
Cytokine production by CD4+ T cell clones. Vaccinia peptides were tested in a dose response assay with their specific T cell clone (2.5×10^4^) in the presence of autologous LCL (5×10^4^) in duplicate wells. Graphs show the cytokine production in response to 5 and 400 ng/ml of peptide (values for background wells were subtracted) at 6 hours and 48 hours post-stimulation. T cell clones were cultured in the presence of autologous LCL (background), vaccinia infected LCL (LCL-Vacc) or PHA. Cytokine production was evaluated by multiplex assay.

The multi-cytokine production was evaluated by a multiplex assay in the supernatants of 6-hour and 48-hour cultures stimulated with peptide at 4 different concentrations (3,000, 400, 40 and 5 ng/ml). Figure 5 shows the cytokine production at 6 hours and 48 hours with 400 ng/ml and 5 ng/ml of peptide. LCL-Vacc or PHA was used as stimuli and control background wells with T cells + uninfected LCL were included. In addition to GM-CSF and TNF-α, most of the clones secreted large amounts of IL-13, IFN-γ, IL-4 and IL-2 in response to 400 ng/ml of peptide and this production could be detected at 6 hours and 48 hours post-stimulation. Interestingly, when low concentrations of peptide were used for stimulation (5 ng/ml), clear differences were observed at the 2 different time-points evaluated. IL-4, IFN-γ and TNF-α were undetectable or produced at very low levels by only 1 of the 4 clones at 6 hours. In addition, at 6 hours post-stimulation IL-2 was detected in the culture supernatants of 3 of the 4 clones and GM-CSF and IL-13 in 2 of the 4 clones. However, in 48 hours supernatants, IL-2 was undetected in 3 of the 4 clones whereas GM-CSF and IL-13 were not only detected in 3 of the 4 clones but were also produced in large quantities (>1000 pg/ml). IFN-γ was only detected upon peptide stimulation (400 ng/ml) but not with LCL-Vacc. In addition, IFN-γ production was detected in response to 5 ng/ml of peptide only for clone VRC19-29. PHA stimulation triggered the secretion of all six cytokines analyzed. The level of production of TNF-α, IFN-γ, IL-4 and IL-2 in response to PHA was similar between 6 and 48 hours probably indicating that there is a maximal cytokine response of the clones to PHA and that response is achieved at 6 hours post-stimulation. This finding is in agreement with kinetics of cytokine gene expression reported by Abdalla et al [25]. In contrast, the level of GM-CSF and IL-13 did increase from 6 hours to 48 hours, showing again that in particular these two cytokines accumulate at high levels in the supernatants of stimulated CD4+ T cell clones. In response to infection (LCL-Vacc), all cytokines, with exception of IFN-γ, were detected in all clones and, similarly to the response to low antigen concentration (peptide at 5 ng/ml), GM-CSF, TNF-α, IL-13 and IL4 were detected at 48 hours at significantly higher levels than those at 6 hours. The multiplex analysis also revealed that clones VRC19-29 and VRC47-38 are more reactive to their specific peptides than the other 2 clones producing higher levels of cytokines at lower peptide concentrations.

### Intracellular cytokine production of vaccinia specific CD4+ T cell clones in response to peptide and vaccinia stimulation

**Figure 6:**
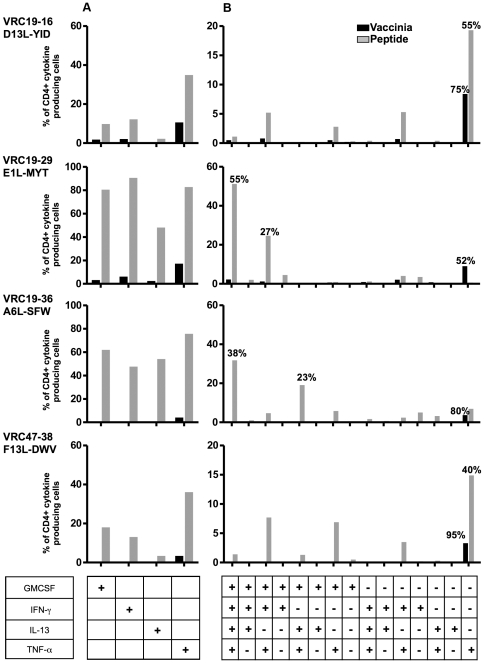
Intracellular cytokine production by vaccinia specific CD4+ T cell clones. Each clonal T cell population was stimulated with 10 µg/ml of its specific vaccinia peptide in the presence of autologous LCL or vaccinia infected LCL for 6 hours. Brefeldin A was added to the cultures after 1 hour of initial incubation and the intracellular cytokine production was measured as described in the *Material and Methods* section. The percentage of CD4+ T cells producing each individual cytokine (A) or any of the 15 possible combinations of cytokines (B) is shown. The largest subset of CD4+ T cells (out of the 15 possible combinations of cytokines) for each clone in response to peptide or vaccinia infected LCL is shown as percentages above the corresponding data bars (B). Results are representative of two experiments.

Intracellular cytokine staining (ICCS) studies were carried out to measure the production of GM-CSF, IFN-γ IL-13, and TNF-α by each CD4+ T cell clone in response to vaccinia infected LCL and to the vaccinia peptides at a single cell level (Figure 6). CD4+ T cell clones were cultured in the presence of LCL-Vacc, peptide-loaded LCL or uninfected LCL for 6 hours. The percentage of cells producing each of the cytokine, irrespective of whether they produce only one or multiple cytokines, is shown in Figure 6A. All clones produced GM-CSF, IFN-γ, IL-13 and TNF-α in response to their specific vaccinia peptide. Clearly, the percentage of cytokine-producing cells is higher in clones VRC19-29 and VRC19-36. Furthermore, TNF-α producing cells in response to LCL-Vacc were detected in all clones, whereas the percentage of cells producing GM-CSF, IFN-γ and IL-13 was either low or not detected. The contribution of the 15 different possible cytokine combinations based on GM-CSF, IFN-γ, IL-13 and TNF-α production to the total cytokine production was assessed for each clone (Figure 6B). This analysis determines the cytokines that were produced by the largest percentage of cells within the overall percentage of cytokine producing cells. For clones VRC19-16 and VRC47-38, the largest percentage of cytokine producing CD4+ T cells in response to peptide (55% and 40% respectively) is represented by cells that only produce TNF-α and no other cytokine (Figure 6B). In contrast, for clones VRC19-29 and VRC19-36 the dominant subset (55% and 38%, respectively) in response to peptide is represented by cells that produce all four cytokines simultaneously. Remarkably, in response to vaccinia stimulation, the largest percentage of cytokine producing CD4+ T cells for all 4 clones is represented by cells that only produce TNF-α and no other cytokine. Overall, these results show that within 6 hours of stimulation, the identified vaccinia peptides induce T cell clones that produce multiple cytokines while in response to vaccinia infected LCL the largest percentage of cells produce only TNF-α.

### CD4+ vaccinia T cell clones are cytotoxic

**Figure 7:**
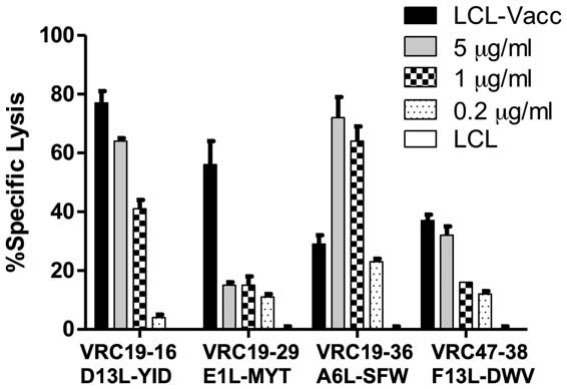
Cytotoxic activity of vaccinia CD4+ T cell clones. Clonal T cells were co-cultured with ^51^ *Cr*-labeled autologous LCL targets (2×10^3^), infected with vaccinia or seeded in the presence of vaccinia peptides at 5, 1 and 0.2 µg/ml. The cytotoxicity activity is expressed as the mean of the percent of specific lysis triggered by the cytotoxic T cell clones after 4 hours at a 30∶1 ratio. These results are representative of three experiments.

It has been previously demonstrated that vaccinia specific CD4+ T cell clones can recognize and kill vaccinia infected targets [26], but there have been few studies focusing on cytotoxic CD4+ T cell responses to smallpox vaccination [17]. The four CD4+ T cell clones identified in this study were tested for their cytotoxic activity against LCL-Vacc and their specific vaccinia peptides. As shown in Figure 7, all CD4+ T cell clones killed vaccinia infected cells. VRC19-16, VRC19-36 and VRC47-38 killed vaccinia peptide loaded LCL in a dose dependent manner and at comparable or higher efficiency than vaccinia infected target cells. VRC19-29 killed peptide loaded LCL at a very low percentage and the killing was not proportional to the amount of peptide tested. The reason for the lack of cytotoxic activity in response to peptide by VRC19-29 is unknown and suggests that some other factors might be present in the vaccinia infected cells and not in the peptide target cells that render the cells susceptible to killing. In general, these results demonstrate that these CD4+ T cell clones are cytotoxic in agreement with the view that CTL-mediated lysis of vaccinia virus may significantly contribute to the effect of the vaccine in controlling the virus infection with poxvirus [17], [27].

### Characterization of the HLA restriction haplotype for the identified peptides

**Figure 8:**
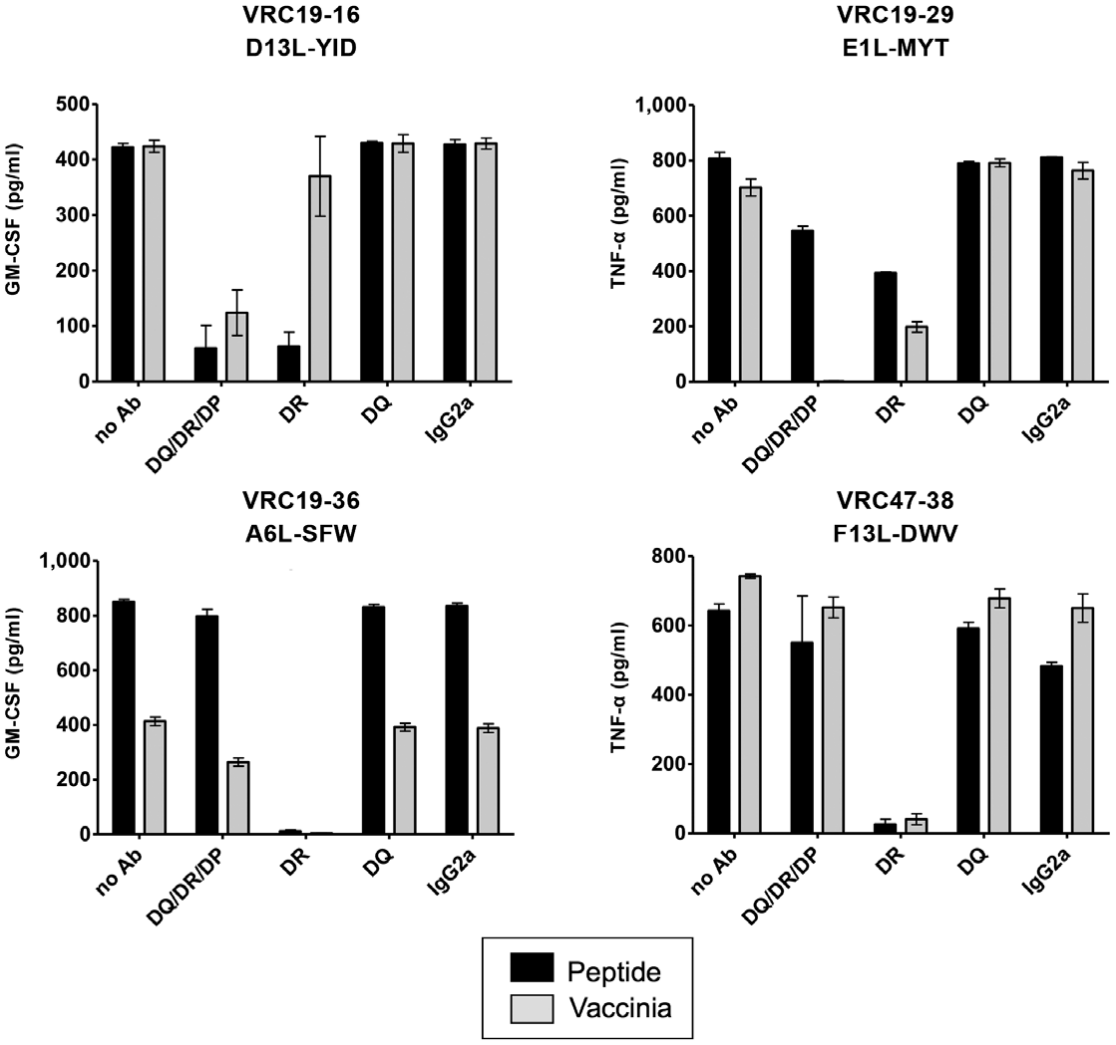
Determination of HLA restriction. Cytokine production for each T cell clone was evaluated following the activation with their specific vaccinia peptides in the presence and absence of HLA blocking antibodies and isotype control anti-human IgG2a. T cells (2.5×10^4^) were seeded with autologous LCL (5×10^4^) and peptide and supernatants were collected after 48 hours of incubation. Data represents averages and standard errors of the mean of TNF-α or GM-CSF concentrations in the supernatant of two replicate culture wells in the presence of 10 µg/ml of antibody and 0.4 ng/ml of peptide, with the exception of peptide A6L-SFW that is shown at 370 ng/ml. Results are representative of two individual experiments.

To determine the HLA restriction context in which the peptides are presented, T cell clones together with uninfected LCL were cultured with peptide in the presence of antibodies that recognize the major HLA loci, namely anti–HLA-DR, anti–HLA-DQ, anti–HLA-DR/DQ/DP or human IgG2a as an isotype control. T cell clones were also tested with LCL-Vacc in the presence or absence of the same antibodies. At least 50% inhibition of GM-CSF and TNF-α production and a consistent pattern in duplicate tests of each mAb was required to assign restriction to any HLA locus. Cytokine production was inhibited for all the clones in response to peptide or vaccinia infection in the presence of antibody anti-HLA-DR (Figure 8). For clones VRC19-29 and VRC47-38 the quantification of GM-CSF production did not reveal any inhibitory effect by any of the antibodies (data not shown). However, inhibition could be observed when TNF-α production was quantified. On the contrary, the inhibitory effect of the antibodies on the peptide stimulated T cell clones VRC19-16 and VRC19-36 was determined from the production of GM-CSF. These results clearly suggest that the 4 peptides are presented to their specific clones in the context of HLA-DR restriction.

To further characterize the DR restriction, B cell transfectants (BLS) cells expressing only 1 single haplotype were used as antigen presenting cells. The available BLS express either the DRB1*1501 or DRB5*0101 haplotype. Since VRC19 donor expresses the DRB1*1501 DRB5*0101, DRB1*0701 and DRB4*0103 alleles, BLS cells expressing DRB1*1501 or DRB5*0101 were used to present the D13L-YID, E1L-MYT and A6L-SFW peptides. As shown in Figure S1, D13L-YID and E1L-MYT peptides are presented in the context of DRB1*1501 to VRC19-16 and VRC19-29 clones, respectively. However, none of these alleles were able to present the A6L-SFW peptide to clone VRC 19-36 indicating that another haplotype than DRB1*1501 DRB5*0101 is used to present the A6L-SFW peptide in donor VRC19.

### Recognition of peptides by vaccinia specific T cell lines

**Figure 9:**
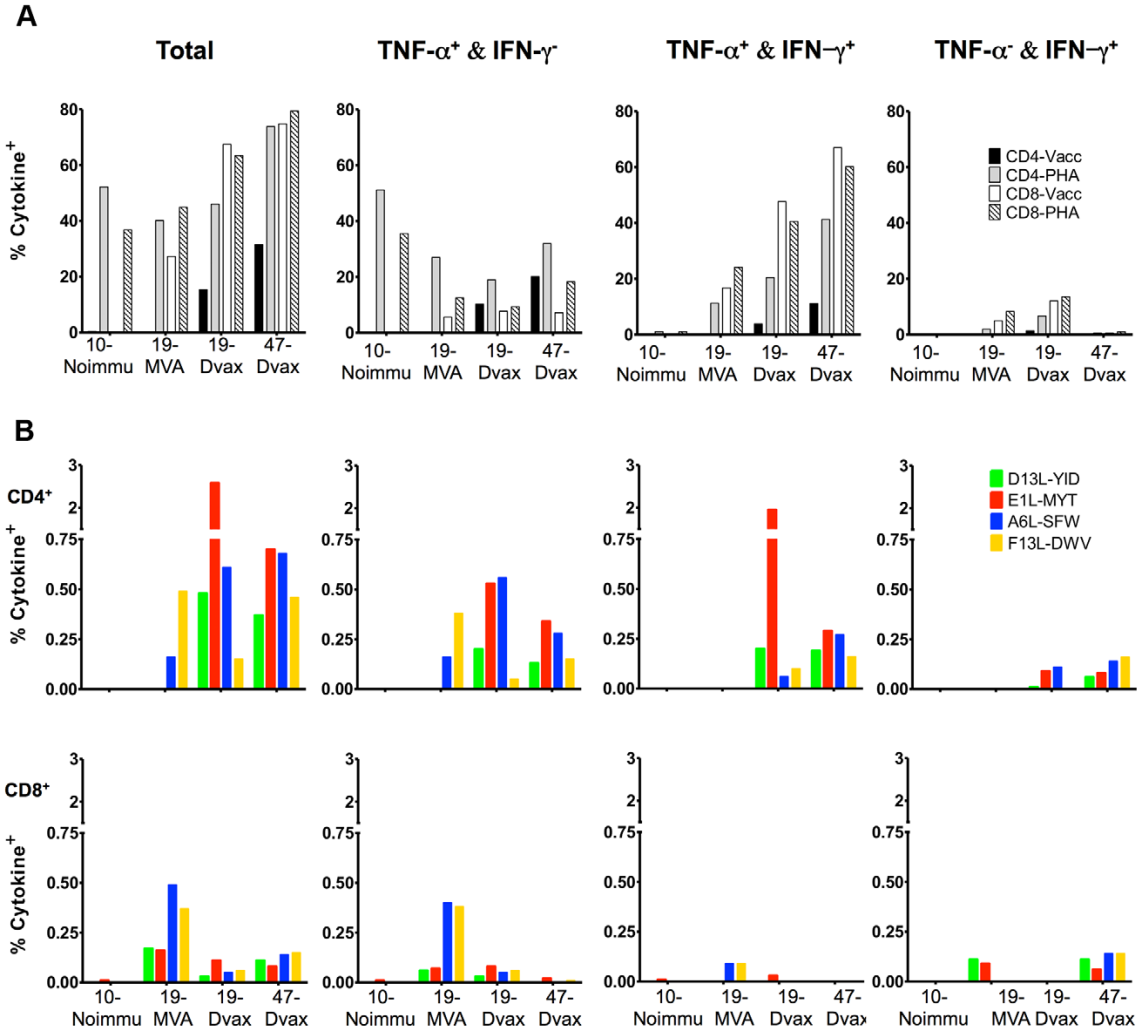
Intracellular cytokine production by vaccinia specific T cell lines. Vaccinia T cells lines were generated with PBMCs from vaccinated donors VRC19 and VRC47, and from unvaccinated donor TPI10 as detailed in the *Material and Methods* section. T cell lines were evaluated for their recognition of vaccinia virus and the vaccinia peptides by intracellular TNF-α and IFN-γ production. Data represent the percentage of CD4+ or CD8+ T cells in each line producing either of the four possible combination of cytokines in response to vaccinia infected autologous LCL and PHA (A) or to the identified vaccinia peptides (10 µg/ml) (B).

To evaluate the recognition of the identified vaccinia CD4+ peptides in bulk populations, short term vaccinia specific T cell lines were derived directly from PBMC from donors VRC19 and VRC47. As noted in the methods, donor VRC47 had been vaccinated with Dryvax once, and donor VRC19 had been first vaccinated with MVA and then boosted with Dryvax. The two time-points samples from donor VRC19, post-MVA (19-MVA) and post Dryvax (19 Dvax), were included. Donor TPI10, who was never vaccinated against smallpox, was included as control. The reactivity to the peptides was analyzed by intracellular staining of IFN-γ and TNF-α production in both CD4+ and CD8+ subsets. Vaccinia stimulation and PHA were used as positive controls. As shown in Figure 9A, analysis in VRC19 donor, after MVA vaccination (19-MVA), showed IFN-γ and TNF-α production by CD8+ T cells but not CD4+ specific responses following vaccinia stimulation (CD4-Vacc and CD8-Vacc subsets). However, both CD4+ and CD8+ subsets clearly responded to PHA stimulation (CD4-PHA and CD8-PHA subsets) demonstrating that the lack of vaccinia CD4+ specific responses was not due to an experimental error or to the absence of a generalized lack of reactivity of the CD4+ population. T cells from this donor upon boosting with Dryvax (19-Dryvax) showed both CD4+ and CD8+ specific T cell responses to vaccinia, suggesting that boosting with Dryvax vaccination clearly expands the CD4+ specific vaccinia responses. Importantly, whereas the CD8+ response to vaccinia in this sample was characterized by cells that produce both TNF-α and IFN-γ cytokines, the CD4+ response was represented by cells that only produce TNF-α or by cells that produce TNF-α and IFN-γ. Similarly, vaccinia specific CD4+ T cells from donor VRC47 upon Dryvax immunization (47-Dryvax) produced either TNF-α, or TNF-α and IFN-γ, but not IFN-γ alone, whereas the CD8+ population produced mostly TNF-α and IFN-γ. As expected, T cells derived from the nonimmunized donor TPI10 responded clearly to PHA stimulation but not to vaccinia infection.

Figure 9B shows the intracellular cytokine response to the peptides. Although vaccinia CD4+ responses were not observed in sample 19-MVA when vaccinia infected LCL were used for stimulation (Figure 9A), clear CD4+ T cell responses to vaccinia epitopes A6L-SFW and F3L-DWV were detected (Figure 9B, top). This response was characterized by TNF-α (+) and IFN-γ (−) producing cells. CD8+ T cells from 19-MVA not only responded to these epitopes but also recognized D13L-YID and E1L-MYT (Figure 9B, bottom). While the CD8+ T cell response to A6L-SFW and F3L-DWV was characterized by cells that produce TNF-α and not IFN-γ, the CD8+ response to D13L-YID and E1L-MYT was represented by cells that produced either TNF-α or IFN-γ alone. Both CD4+ and CD8+ T cells derived from 19-Dryvax and 47-Dryvax samples responded to all 4 vaccinia epitopes, but CD8+ T cells showed a lower percentage of cytokine producing cells. In both donors, after Dryvax vaccination, the CD4+ response to the peptides was characterized by cells that produced either TNF-α or TNF-α and IFN-γ, but not IFN-γ alone. T cells from unvaccinated TPI10 control subject did not respond to any of the vaccinia epitopes identified in this study. Together, the results shown here demonstrate that vaccinia specific responses can be detected in vaccinated donors using the vaccinia epitopes identified in this study and highlight the importance of evaluating TNF-α as well as IFN-γ production in order to determine the CD4+ specificity of the smallpox vaccination response in humans.

## Discussion

During the past 10 years significant efforts have been focused on the understanding of the immunity and immunological memory to vaccinia virus and other poxviruses (reviewed in ref. [28]. Particularly, it has been shown that both, CD8+ and CD4+ T cells respond vigorously following smallpox vaccination of humans [4]. The kinetic analysis of CD4+ versus CD8+ responses suggests that the CD4+ response is lower than the CD8+ at 2 weeks post-vaccination [2], but similar in magnitude at 1 month post-vaccination [2]–[4]. Furthermore, both vaccinia specific neutralizing antibodies and CD4+ responses are detected in subjects after more than 40 years of Dryvax immunization [4]–[6] or variola infection [5], [7]. In a murine model of vaccinia infection it has been recently demonstrated that there is a strong concordance between CD4+ T cells and antibody protein targets. In fact, this study demonstrated that 11 out of the 18 proteins recognized by CD4+ T cell responses were also detected by antibodies [8]. It is also well established that CD4+ T cells are important in primary clearance of vaccinia and in the induction and maintenance of long-term memory and protection from variola challenge. Importantly, Puissant-Lubrano in a recent study showed that the number of residual vaccinia-specific CD4+ lymphocytes (but not CD8+) is inversely associated with the size of the skin lesion formed in response to revaccination in humans [29]. Together, these findings clearly support the importance of CD4+ T cells in long term memory to vaccinia infection.

The elucidation of antigen specificity in response to vaccinia immunization has been more difficult for CD4+ than for CD8+ T cells. This could be explained in part by the fact that most CD8+ T cell epitopes have been identified using prediction binding algorithms which have relatively poor prognostic ability for prediction of peptides that bind class II molecules [9], [10]. In addition, the use of traditional readouts of CD8+ specificity, such as IFN-γ detection to characterize CD4+ T cell responses, could have underestimated the frequency of CD4+ responses and therefore precluded the understanding of CD4+ specificity. With this in mind, we hypothesized that a comprehensive characterization of the CD4+ T cell response to vaccinia could reveal better readouts of specificity to enumerate and characterize responses that otherwise would have not been detected. In this study, we implemented an approach in which PBMC from human immunized donors were a single time *in vitro* stimulated with vaccinia virus, and the resulting CD4+ T cell lines and clones were characterized in terms of their cytokine production (22 cytokines, Figure 1). Positive responses were observed for 14 of the 22 cytokines tested, namely GM-CSF, IFN-γ, Il-2, IL-3, IL-4, IL-5, IL-6, IL-8, IL-10, IL-12(p40), IL-13, IP-10, Rantes and TNF-α. Six of these cytokines (GM-CSF, IFN-γ, IL-2, IL-4, IL-13, TNF-α) were selected to characterize both the vaccinia and the peptide responses by CD4+ T cells and all were found to be positive. Our results show that the agonistic activity of the peptides and the response to vaccinia infection is enough to induce a large spectrum of cytokines. Indeed, ICCS revealed that both Th-1 and Th-2 cytokines are being produced simultaneously by the same clonal cells in response to peptide, demonstrating that the clones cannot necessarily be assigned to any particular CD4+ Th-subset. Furthermore, all 4 vaccinia CD4+ T cell clones showed the capacity to kill vaccinia infected targets and vaccinia peptide loaded APCs. However, the concentration of antigen required to trigger cytotoxic T cell responses are higher than those needed to induce cytokine production. Together, our studies show that the vaccinia clones studied here are poly-functional and suggest that the detection levels of each of those functions depend on the amount of antigen used for stimulation.

The detection of GM-CSF in response to mixtures of a positional scanning library was found to be an optimal and reliable readout of CD4+ activation and led to the identification of 4 novel immunogenic vaccinia epitopes derived from proteins prevalently recognized by CD4+ T cells and antibodies upon smallpox vaccination in humans. Previously, GM-CSF was measured in the serum of vaccines in an effort to correlate adverse effects with the presence of cytokines [30], [31] but, to our knowledge, this is the first time that GM-CSF is monitored and is shown to be clearly produced by T cells in response to vaccinia.

The determination of cytokine production at decreasing concentrations of peptide clearly showed that the production of GM-CSF requires less peptide than TNF-α (Figure 4). This finding explains why GM-CSF and not TNF-α was detected in response to mixtures of the positional scanning library composed of billions of different peptides present at very low concentrations. To our knowledge, this study shows for the first time that lower antigen concentrations are required for substantial production of GM-CSF as compared to other cytokines. Its detection after 6 hours and its increase after 48 hours (Figure 5) are in agreement with a report by Abdalla et al in which mRNA of GM-CSF is detectable as fast as 30 minutes after stimulation of CD4+ T cells with a recall antigen (purified protein derivative) and it is maintained at high levels over a period of 96 hours [25]. Furthermore, no other evaluated cytokine (IL-2, IL-5, IFN-γ, TNF-α) maintained the fold increase observed for GM-CSF. Topalian et al, also suggested the lower antigen concentration requirement for GM-CSF production [32]. In this study, CD4+ T cells produced GM-CSF at significantly higher levels than TNF-α, IL-4, and IFN-γ in response to tumor lysates. While GM-CSF is not a standard cytokine used for monitoring human T cell responses against vaccines or infection, its production by activated T cells is generally accepted. A review by Shi et al in 2006 describes its immunobiology specifically in T cells [33], and more recently a number of studies have demonstrated the advantages of monitoring T cells expressing GM-CSF in the context of infection [34], [35], and vaccine responses to bacterial [36] and tumor antigens [37].

Similar results were obtained either by ICCS (Figure 6) or by measuring the cytokines in the culture supernatants (Figure 5) when the T cell clones were stimulated with their specific vaccinia peptides for 6 hours. In contrast, in response to vaccinia infection (LCLs-Vacc) ICCS revealed that the large majority of the cytokine producing cells produce only TNF-α (52% to 95% depending on the clone) whereas measurement of the same cytokines in the culture supernatants show that GM-CSF, IL-13 and TNF-α are clearly detected. It is therefore important to note that different readouts of activation, antigens, and times of stimulation provide multiple snapshots of the same dynamic process. Indeed, the importance of monitoring multiple cytokines when analyzing the T cell response to vaccination and infection has been previously reported. However, in response to vaccinia very few studies have looked at cytokines other than IFN-γ production. Hammarlund et al [4] monitored TNF-α and IFN-γ producing vaccinia specific T cells by ICCS, and Ryan et al [38] studied the production of 11 cytokines in response to vaccinia, but they did not include TNF-α, GM-CSF and IL-13 which we identified in the present study to be clearly produced by vaccinia specific T cell clones. In addition, it is clear that a significant percentage of peptide and vaccinia specific T cells in bulk populations can only be detected by ICCS by the measurement of TNF-α production (Figure 9A and 9B). Together, these results may explain the intriguing observation [17] that in bulk PBMC cultures, the total numbers of IFN-γ secreting cells responding to the individual peptides is higher than the number of IFN-γ secreting cells responding to whole vaccinia virus. Our findings support the idea that quantifying only IFN-γ producing cells underestimates the CD4+ T cell response to vaccinia virus. Furthermore, Jing et al showed that CD4+ responses to a group of vaccinia fragments were considered positive by proliferation but negative by IFN-γ quantification [14]. In addition, results with dengue virus also showed that the majority of CD4+ cytokine-positive T cells from donors immunized with the live attenuated vaccine produced either TNF-α alone or TNF-α and IFN-γ when stimulated with heterologous antigen serotypes [39].

The results presented here show that human vaccinia specific CD4+ T cell clones that emerge following smallpox vaccination produce high levels of IL-13 in response to vaccinia and peptide stimulation. Furthermore, vaccinia specific T cell lines and ex-vivo responses in PBMC from vaccinated donors also respond with IL-13 secretion upon *in vitro* vaccinia stimulation (Figure 1 and data not shown). The role of IL-13 on vaccinia infection has been studied in the context of atopic dermatitis and its effect on vaccinia growth in keratinocytes has been reported [40], [41]. In addition, it has been shown that following smallpox immunization [2] a small proportion of vaccinia specific CD4+ T cells produce exclusively IL-13 during the peak effector phase of the response (2 weeks following Dryvax vaccination). Huaman et al showed that in humans, immunization with a fragment of *Plasmodium falciparum* triggers memory CD4+ T cells that produce IL-13 [42]. In this regard, it has been suggested that the production of an appropriate amount of IL-13 during infection could moderate the degree of pathogen-induced inflammation. Particularly, data generated in a rat model of parainfluenza type 1 (Sendai) virus infection indicated that appropriate secretion of IL-13 could also serve to limit the extent of virus-induced inflammation [43]. Whether IL-13 has a role in long term protection upon vaccination cannot be concluded from these results, but it is clear that quantification of IL-13 secretion by CD4+ T cells could be used to follow up and characterize vaccinia responses upon vaccination.

It is interesting that although all the clones produced IFN-γ in response to PHA and peptide, it was not detected in response to vaccinia infected LCLs when measured in the culture supernatants upon 48 hours of stimulation. In addition, all the clones showed IFN-γ production when measured by ICCS upon peptide stimulation and at a lower level upon vaccinia stimulation (Figure 6, VRC19-16 and VRC19-29 clones). The most likely explanation for the lack of correlation on the intracellular detection of IFN-γ, but not in the supernatant cultures upon vaccinia stimulation, is the expression of IFN-γ binding proteins (IFN-γ BPs) by vaccinia virus. IFN-γ BPs have been reported to efficiently bind and antagonize soluble IFN-γ [44], [45]. An alternative explanation derives from studies reported by Zaunders et al in which they show that IFN-γ production is characteristic of effector CD4+ T cells at very early time-points upon vaccination, but IFN-γ levels decrease as much as 10-fold lower than other indicators of vaccinia specificity during memory T cell differentiation [46].

In this study vaccinia specific CD4+ T cell clones from human immunized subjects were screened with combinatorial peptide libraries. The screening data was then integrated by a computational analysis, known as positional scanning based biometrical analysis [20], with a vaccinia specific protein database for the prediction of stimulatory peptides. Positional scanning libraries have been extensively and successfully used for the identification of T cell epitopes in a broad range of human diseases [47]–[50]. The positional scanning library profile for each of the vaccinia clones revealed that the amino acids of the identified peptides in most of the positions correspond to the defined amino acids of the mixtures with the highest stimulatory potency (shown in Table S1). The high correspondence on amino acids from active mixtures in the active peptides identified for the vaccinia clones is similar to previous results obtained with other pathogen specific T cell clones [51] and confirms that the use of positional scanning libraries together with the biometrical analysis provides an efficient unbiased methodology for epitope and antigen identification.

Our previous studies on T cell pathogen specificity have shown that more than one peptide can be recognized by a single pathogen specific T cell clone [47], [49], [52]. However, this was not the case in this work in which only one vaccinia epitope was found to stimulate each of the clones. A possible explanation for this finding is that we have used a vaccinia virus (Western Reserve) protein database instead of the complete viral database (Vaccinia: 216 proteins and 59,892 decapeptides versus Viral Genpept 156: 506,176 proteins and 125,055,759 decapeptides). Alternatively, the vaccinia clones presented here are intrinsically not highly cross-reactive and thus, independent of the database used for the biometrical analysis, there is only one (the peptides identified in this study) or very few optimal peptides capable of triggering their activation. Indeed, the biometrical analysis of the viral database revealed, for 3 of the 4 clones, that the identified vaccinia peptide in this work ranked within the top 30 peptides for the 125 million peptides scored (data not shown). Although all these peptides were not synthesized and tested, and thus their possible agonistic activity has not been determined, this finding strongly suggests that the clones are highly specific for the antigens reported here.

Importantly, the epitopes identified by positional scanning peptide libraries and their corresponding proteins are by definition immunogenic, since they have been derived by interrogating vaccinia specific T cell clones expanded in vivo during exposure to the vaccine, and hence relevant to the immune response. Other groups have also reported the clear advantages of using “T cell driven” approaches for the identification of antigens recognized by large size pathogens specific T cells. The T cell driven approach resulted in the identification of immunodominant epitopes for *Mycobacterium tuberculosis* with a genome encoding for approximately 4,000 proteins [53]. The methodology implemented in our study used a single *in vitro* vaccinia virus stimulation of PBMC derived from smallpox immunized subjects in order to expand and clone vaccinia specific T cells. While it is possible that this stimulation may result in the expansion of cross-reactive T cells that were not originally triggered by the *in vivo* vaccinia immunization, our data shows that vaccinia specific T cells are only expanded in vaccinia immunized donors and not in unvaccinated subjects (Figure 9 and data not shown) indicating that the one time *in vitro* expansion with the virus expands the repertoire triggered by immunization.

Four novel vaccinia epitopes and their corresponding vaccinia antigens (Table 1) recognized by CD4+ T cells from humans immunized with the smallpox vaccine are presented in this study. D13L-YID and F13L-DWV proteins are from late expression membrane proteins, E1L-MYT from an early expression enzyme, and A6L-SFW is from a protein with unknown function and late expression. All four proteins have 100% homology within vaccinia and variola strains. In addition, and in agreement with recent findings by Jing et al showing that HLA-DR dominates the presentation of vaccinia antigens to CD4+ T cells [15], all four identified epitopes in this study were found to be HLA-DR restricted. The identification of the epitopes presented here used a T cell driven approach that has not been previously utilized to identify immunogenic pathogen proteins following vaccination in humans. This approach elucidates the peptides and viral antigens recognized by virus specific T cells using an unbiased collection of peptides (positional scanning libraries) presented by autologous LCLs. Thus, no previous knowledge about MHC restriction or antigen specificity is required.

To evaluate the performance efficacy of the T cell driven strategy presented here we compared the extent of recognition by antibodies and CD4+ T cells of each of the 181 vaccinia proteins derived from all reported human studies. Using separately the information from CD4+ T cell [13] or antibody responses [13], [54]–[57], we calculated a percentage of recognition for each vaccinia protein by dividing the number of subjects that showed protein recognition by the total number of subjects tested (76 or 126 for antibodies and 11 for T cells). Based on the overall distribution of the percentages of recognition and the number of subjects tested we established a cutoff of >40% and >10% for CD4+ T cell or antibody responses, respectively for a protein to be considered predominantly recognized. Using these thresholds 31 proteins were determined as predominantly recognized by CD4+ T cells and 21 by antibodies (data not shown, manuscript in preparation). Table 2 shows the function, temporal expression [58] as well as the percentage of recognition for each of the 10 proteins (4 reported here and manuscript in preparation) that we have identified using T cell driven approaches. Strikingly, 6 out of the 10 identified proteins (D13L, A7L, F13L, A6L, A10L and H5R) are within the 31 predominantly recognized proteins by CD4+ T cells (percentage of recognition >40%) and 5 (D13L, F13L, H5R, A10L and L1R) are within the 21 predominantly recognized proteins by antibodies (percentage of recognition >10%). The significance of our results can be statistically analyzed by comparing the probability of these findings to random sampling. If a random sample of 10 proteins were selected from the 180 proteins analyzed by CD4+ T cell responses, the probability that 6 or more would be among the 31 predominantly recognized proteins would be only 0.21%. Similarly, if a random sample of 10 proteins were selected from the 181 proteins analyzed by antibody responses, the probability that 5 or more would be among the 21 predominantly recognized proteins would be 0.23%. The percentage of predominantly recognized proteins that we identified is about 60%, which is clearly larger than the random probabilities, and indeed the above probabilities indicate that the method used in the present study yields results significantly better than random selection. These findings clearly reveal that the T cell driven approach used in the present study is very efficient in the identification of immunogenic and predominantly recognized proteins. Since the analysis of the whole pathogen proteome to identify relevant antigens might not be feasible for larger pathogens (>1,000 proteins), there is a clear advantage in using positional scanning libraries to elucidate the antigen specificity of the pathogen specific T cells.

**Table 2:** Recognition of vaccinia proteins for which peptides have been identified using the “T cell driven” approach presented in this study.

Protein	Function, temporal expression^a^	Proliferative responses (11 subjects)^b^	Antibody recognition (76 or 126 subjects)^c^	Other human CD4 epitopes reported, Method
D13L	Rifampicin target/membrane, L	100%	60%	Yes, recombinant fragments
A10L	precursor p4a of core protein 4a, L	100%	60%	Yes, MHC elution and DR1 binding prediction
A7L	82 kDa large subunit of early gene transcription factor VETF, L	64%	1%^d^	Yes, recombinant fragments and DR1 binding prediction
F13L	EEV membrane protein, L	64%	15%	No
A6L	Unknown, L	55%	0%^d^	No
H5R	Morphogenesis related, Transcription factor VLTF-4, E	55%	16%	No
D1R	mRNA capping enzyme, E	27%	7%^d^	Yes, overlapping peptides and DR1 binding prediction
E1L	Poly-A polymerase, E	18%	0%	No
L1R	IMV membrane protein, L	18%	37%	Yes, recombinant fragments and overlapping peptides
A28L	Unknown, putative signal peptide, L	0%	0%	DR1 binding prediction

a Protein Temporal expression derived from Assarsson et al [*ref*.58]. L (late) and E (early).

b Percentage of subjects (out of 11) that showed proliferative response to the recombinant antigen [*ref*.13].

c Percentage of subjects (out of 76 or 126) that showed antibody recognition to the recombinant antigen derived from 5 different studies [refs. 13], [54]–[57].

d Proteins tested with 76 subjects.

In conclusion, we describe a methodology that can be implemented and applied to determine the specificity of the T cell response upon vaccination or infection with large size pathogens for which other methodologies would be more limiting. Together, the single pathogen *in vitro* stimulation, the selection of CD4+ T cells specific to the pathogen by limiting dilution, the evaluation of pathogen specificity by detecting multiple cytokines (including GM-CSF), and the screening of the clones with synthetic combinatorial libraries constitutes a novel and valuable approach for the elucidation of the CD4+ T cell specificity in response to large pathogens in human samples. We believe that further efforts on the development of miniature T cell activation assays will be extremely beneficial to reduce the number of clonal CD4+ T cells required to test combinatorial peptide libraries and to be able to define the spectrum of specificities with higher throughput.

Finally, our results support the notion that the cytokines selected to profile CD4+ T cell specific responses upon infection or vaccination should not rely only in IFN-γ secreting cells, but on the assessing of multi-cytokines at different time-points and by using different readout technologies. We believe that the vaccinia antigens identified in this study contribute to the knowledge base of the human immune response to vaccinia immunization and could lead to the development and evaluation of novel and safer smallpox vaccines.

## Materials and Methods

### Donors

Peripheral blood mononuclear cells (PBMC) from modified vaccinia Ankara (MVA) and/or Dryvax-immunized donors were made available from a clinical study carried out at the Vaccine Research Center, NIH [22]. These studies were approved by and performed in compliance with the guidelines of the National Institute of Allergy and Infectious Diseases Institutional Review Board, and were performed in accordance with 45 CFR Part 46, U.S. Food and Drug Administration regulations. All subjects signed written informed consent documents. Subject VRC19 received a single injection of MVA and a subsequent challenge inoculation of Dryvax. The PBMC samples for subject VRC19 used in this study were obtained 2 months after MVA immunization (19-MVA) and 4 months after MVA and 1 month after Dryvax (19-Dryvax). Subject VRC47 received two placebo injections and one Dryvax immunization. The VRC47 samples used in this study were drawn 1 and 3 months after Dryvax vaccination. The sample obtained after 1 month of vaccination was used to derive the VRC47-38 T cell clone whereas the sample drawn 3 months post-vaccination was used to generate a vaccinia line for testing the recognition of the peptides (47-Dryvax, Figure 9). The HLA class II genotypes for each donor are as follows: VRC19: DRB1*0701, 150101; DRB4*0103; DRB5*0101; DQB1*0202, 0602; DPB1*0401, 2701; DQA1*0102, 0201; DPB1*8501 and VRC47: DRB1*0701; DRB4*0101; DQB1*0202; DPB1*0301, 0501; DQA1*0201.

### Vaccinia specific T cell lines

Isolated PBMC from donors VRC19 and VRC47, samples 19-Dryvax and 47-Dryvax respectively, were stimulated with WR vaccinia virus at a multiplicity of infection (MOI) of 0.1. When cells were actively dividing (day 5–8) they were immortalized using a murine leukemia virus based retroviral vector carrying the telomerase gene (hTERT) [59]. This vector preferentially transduces cells that are dividing, and the procedure results in the selective transduction of cells that are proliferating in response to antigen stimulation. Introduction of hTERT allows for long-term culture and production of large numbers of T cells while maintaining MHC-restricted antigen-specific reactivity and T cell functions. Lines were frozen and thawed when needed. After thawing, lines were expanded by allogeneic stimulation using the mitogen phytohaemagglutinin (PHA) (Remel, Inc) at 0.5 µg/ml in the presence of irradiated, allogeneic feeder cells and 100 IU/ml of IL-2.

### Generation of vaccinia specific T cell clones

The VRC19 and VRC47 vaccinia stimulated and immortalized T cell lines described above were cloned by limiting dilution. PHA allogenic stimulated cells were plated in wells at different cell concentrations ranging from 10 to 0.1 cells/well. After one or two stimulations, depending on the growth, a fraction of the cells were tested for vaccinia reactivity as described below. Cells that responded to vaccinia infected antigen presenting cells and grew to large numbers were characterized for CD4, CD8 and then by TCR Vβ expression by flow cytometry or PCR. Cell cultures that stained with one TCR Vβ antibody were considered T cell clones (TCC). Those that were negative for any of the antibodies were further tested by PCR to confirm clonality.

### Vaccinia virus infection of antigen presenting cells

The antigen presenting cells used in functional assays to determine the vaccinia reactivity of vaccinia lines and clones were Epstein-Barr virus (EBV)-transformed autologous lymphoblastoid cell lines (LCL). LCL were derived from PBMC using EBV isolate B95.8 [60]. Cryopreserved vaccinia virus (Western Reserve) was used at a multiplicity of infection (MOI) of 0.1 for the infection of LCL. To infect the LCL, cells were pre-incubated with virus at a concentration of 10×10^6^ cells/ml for 1 hour. Then the cells were diluted at 1×10^6^ cells/ml and incubated overnight at 37°C, 5%CO_2_.

### Vaccinia reactivity test

T cell lines or clones (25×10^3^ cells per well) were cultured alone, stimulated with PHA (0.5 µg/ml) or in the presence of infected or uninfected LCL (50×10^3^ cells per well) in 96-well U bottom plate at a final volume of 250 µl of Opti-MEM Reduced Serum Medium (Invitrogen, Carlsbad, CA). After 48 hours, supernatants were collected and GM-CSF and TNF-α production was quantified by ELISA.

### Positional scanning decapeptide library and individual peptides

Both positional scanning libraries and individual peptides were synthesized using the simultaneous multiple peptide synthesis technology [61]. The decapeptide library used in this study is a synthetic N-acetylated, C-terminal amide, L-amino acid combinatorial peptide library arranged in a positional scanning format [62]. It consists of 200 mixtures in the OX9 format where O represents one of the 20 natural L-amino acids in a defined position and X represents all of the natural amino acids, with the exception of cysteine, in each of the remaining positions [62]. For example, the first mixture has alanine (A) in position 1 (A1X9) whereas mixture number 200 has tyrosine (Y) in position 10 (X9Y10). Each OX9 mixture consists of 3.2×10^11^ (19^9^) different decapeptides in approximate equimolar concentration, and the total ×10 library consists of 6.4×10^12^ (20×19^9^) different peptides. Assuming an average molecular weight of 1200 for a decapeptide mixture and a concentration of 100 µg/ml (83 µM), the concentration of each individual peptide is 2.6×10^−16^ M. For the individual peptides, synthesized for each clone, the purity and identity were characterized by liquid chromatography and mass spectrometry.

### Library screening and biometrical analysis

Clonal T cells were cultured (25×10^3^ cells per well) together with LCL (50×10^3^ cells per well) in microtiter plates in standard T cell medium containing each of the mixtures of the library at 200 µg/ml. Medium used for cultures consisted RPMI 1640 (Fisher Scientific, Pittsburgh, PA) supplemented with 10% human serum (Gemini Bio Products, West Sacramento, CA), 1% HEPES buffer (Fisher Scientific, Pittsburgh, PA), 2-βME at 50 µM, 1% penicillin-streptomycin (Fisher Scientific, Pittsburgh, PA), 1% glutamine (Fisher Scientific, Pittsburgh, PA), 1% sodium pyruvate (Fisher Scientific, Pittsburgh, PA) and 1% non-essential amino acids (Fisher Scientific, Pittsburgh, PA). Culture supernatants were harvested at 48 hours and GM-CSF production was determined by ELISA. The positional scanning based biometrical analysis was carried out as previously described [20]. Briefly, a positional scoring matrix was generated by assigning a value of the stimulatory potential to each of the 20 defined amino acids in each of the ten positions of the decapeptide library. Based on a model of independent contribution of individual amino acid to peptide antigen recognition, the predicted stimulatory score of a given peptide is the sum of the stimulatory potential of all amino acids contained in the peptide in each position. Using a web-based search tool [21] the scoring matrix was applied to rank, according to their stimulatory score, all the naturally overlapping 10-mer peptides in the protein sequences within the vaccinia virus (Western Reserve) protein database. For the biometrical analysis of clones 19-16 and 19-29 the average of the results of the positional scanning library data obtained from 3 or 5 different screenings, respectively, was used to generate their matrix. Using this matrix a list of predicted peptides for each clone was generated and the top 35 peptides for clone 19-16 and the top 18 peptides for clone 19-29 were selected for synthesis. For clones 19-36 and 47-38, the range of GM-CSF production in response to the mixtures of the positional scanning library varied significantly between the different screenings precluding the calculation of an average for the generation of the matrix. Therefore, a different strategy was used in which a biometrical analysis for each of the screenings was carried out. Each screening (2 for clone 19-36 and, 3 for clone 47-38) was used to build a matrix and to derive a list of predicted peptides. Fifteen peptides for clone 19-36 were selected which included the top 5 peptides from each screen and, the top common peptides within the first 40 top ranked peptides in each of the screens. For clone 47-38, 21 peptides were selected for synthesis which included the common peptides between the top 50 peptides generated in each of the 3 screenings. All synthesized peptides were tested at 10 and 1 µg/ml with their respective T cell clones for stimulatory activity based on GM-CSF and TNF-α release.

### Cytokine detection. ELISA, Multiplex and ICCS

The assessment of GM-CSF and TNF-α production was performed using ELISA kits according to the manufacturer (BD Pharmingen, San Diego, CA). Linear regression standard curves were used to convert O.D. 492 nm values to specific cytokine concentrations using Prism. Multiplex cytokine detection was performed with Milliplex MAP kit (for 6 or 22 cytokines) following the manufacturer's directions (Millipore, St. Charles, MO) and Luminex instrument and Beadlyte software were used for analysis. For intracellular cytokine staining (ICCS) peptides (10 µg/ml) were added to 3×10^5^ clonal T cells or 5×10^5^ T cell lines in 200 µl T cell media for 6 hours. A total of 0.6–1×10^6^ autologous LCL were added as APC. Brefeldin A was added for 5 hours. Each sample was stained with anti-CD4 and anti-CD8, fixed, permeabilized and then stained with anti-IFN-γ and anti-TNF-α. Background controls were T cell clones or lines with LCL and without peptide addition. Positive controls were T cell clones or lines stimulated with PHA.

### HLA restriction

For HLA restriction studies, T cell clones (TCC) together with uninfected LCL were cultured with peptide in the presence of 10 µg/ml of the following antibodies; anti–HLA-DR (BD Biosciences), anti–HLA-DQ (BD Biosciences), anti–HLA-DR/DQ/DP (BD Biosciences) or human IgG2a (BD Biosciences) as an isotype control. TCC were also tested in the presence of vaccinia infected LCL in the presence or absence of the same antibodies listed above. GM-CSF and TNF-α were measured after 48 hours incubation. Further characterization of the HLA-DR restriction for donor VRC19 was performed using BLS cells as antigen presenting cells. Briefly, BLS cells were incubated with peptide for 1 hour at 37C and then washed and cultured with the corresponding TCC for 48 hours.

### Cytolytic activity assay

The cytolytic activity of the vaccinia specific clones was determined at various effector-to-target (E∶T) ratios in a 4 hours standard CTL assay. Briefly, peptide-pulsed-, vaccinia infected- or untreated- autologous LCL labeled with ^51^Cr were used as target cells. Target cells were incubated with each of the clones in a 96-well plate. Wells with only labeled target cells were used to establish the spontaneous ^51^Cr release whereas wells containing 2.0% igepal CA 630 and target cells were added to determine the maximum release. After 4 hours incubation, 75 µl of supernatant was transferred to a flexible plate containing 200 µl of scintillation fluid. Plates were counted on a Trilux 1450 Microbeta liquid scintillation counter. Specific percent lysis was calculated as follows: ((experimental release-spontaneous release)/(maximum release-spontaneous release) ×100).

## Supporting Information

Figure S1 **HLA restriction of peptides and vaccinia recognition determined using single haplotype expression BLS cells.** VRC-19 clones were cultured in the presence of BLS transfected cells expressing either the DRB5*0101 (DR2a) or DRB1*1501 (DR2b) haplotypes and their respective specific peptide at the indicated concentrations. Cultures including autologous LCL or vaccinia infected LCL were used as controls. The production of TNF-α and GM-CSF detected in the supernatants of duplicate wells after 48 hours of stimulation is shown.
(PDF)

Table S1 **Matrices derived from screening of decapeptide positional scanning library with clones VRC19-16, VRC19-29, VRC19-36, and VRC47-38.**
(PDF)

Table S2 **Peptides identified from positional scanning based biometrical analysis for each T cell clone.**
(PDF)
