# Supplementary figures and images for: GM-CSF Production Allows the Identification of Immunoprevalent Antigens Recognized by Human CD4+ T Cells Following Smallpox Vaccination
Source: PLoS One. 2011 Sep 9;6(9):e24091. doi: 10.1371/journal.pone.0024091 (PMC3170313; doi:10.1371/journal.pone.0024091)

**VRC19-16**  
**D13L-YID**

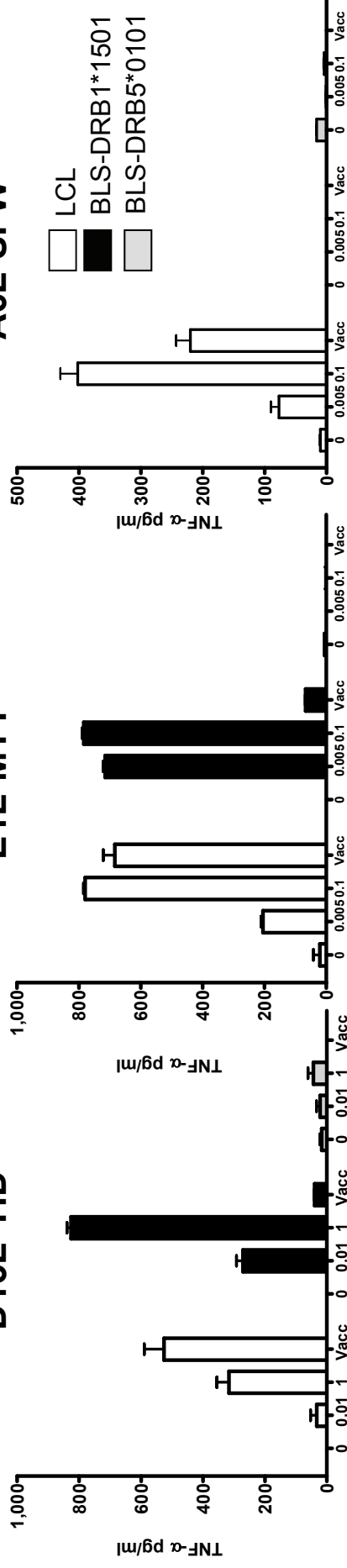

**VRC19-29**  
**E1L-MYT**

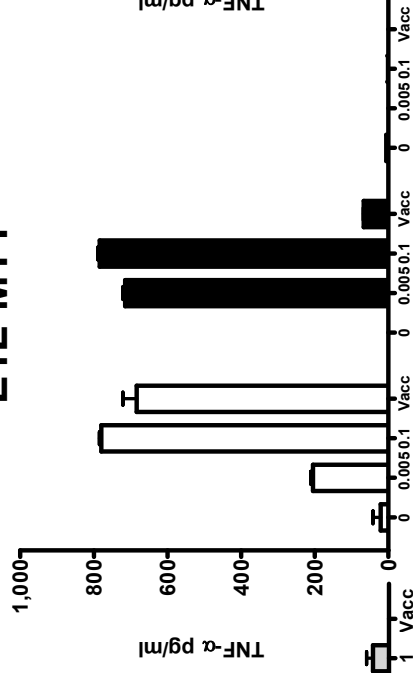

**VRC19-36**  
**A6L-SFW**

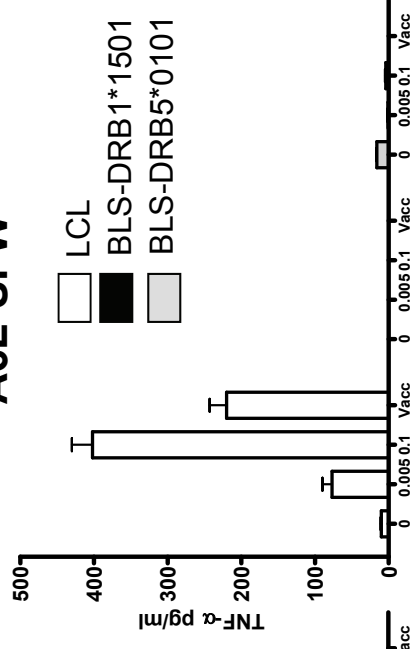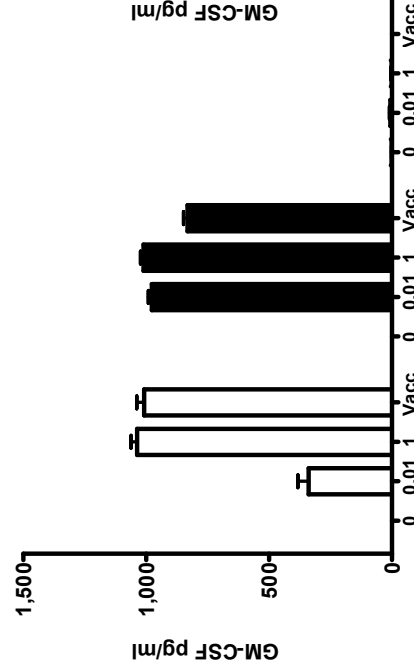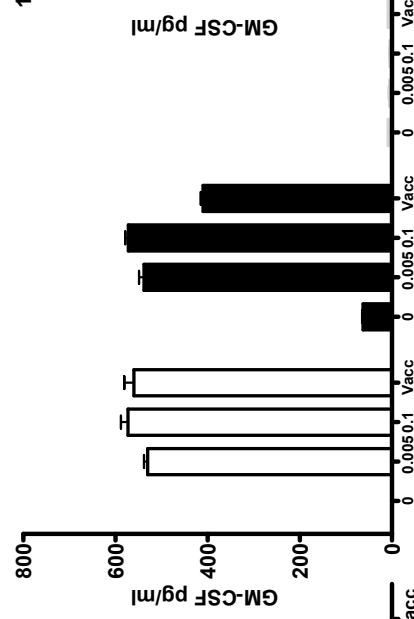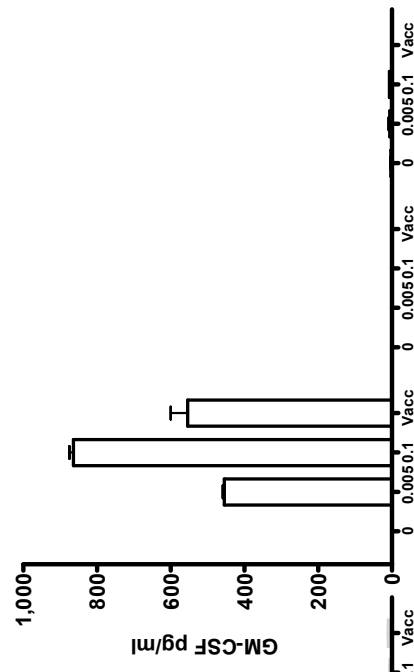

Supplement: Figure S1 — HLA restriction of peptides and vaccinia recognition determined using single haplotype expression BLS cells. VRC-19 clones were cultured in the presence of BLS transfected cells expressing either the DRB5*0101 (DR2a) or DRB1*1501 (DR2b) haplotypes and their respective specific peptide at the indicated concentrations. Cultures including autologous LCL or vaccinia infected LCL were used as controls. The production of TNF-α and GM-CSF detected in the supernatants of duplicate wells after 48 hours of stimulation is shown. (PDF) [file pone.0024091.s001.pdf]
