# Supplementary material for: GM-CSF Production Allows the Identification of Immunoprevalent Antigens Recognized by Human CD4+ T Cells Following Smallpox Vaccination
Source: PLoS One. 2011 Sep 9;6(9):e24091. doi: 10.1371/journal.pone.0024091 (PMC3170313; doi:10.1371/journal.pone.0024091)
Supplement: Table S1 — Matrices derived from screening of decapeptide positional scanning library with clones VRC19-16, VRC19-29, VRC19-36, and VRC47-38. (PDF) [file pone.0024091.s002.pdf]

Table 1S. Matrices derived from screening of decapeptide positional scanning library with clones VRC19-16, VRC19-29, VRC19-36 and VRC47-38

VRC 19-16 — Score D13L$_{283-292}$ YIDAYVSRLL= 1526

	P1	P2	P3	P4	P5	P6	P7	P8	P9	P10
**A**	14	32	20	**211**	23	13	24	0	16	17
**C**	114	138	44	0	76	1	2	4	53	55
**D**	12	25	**91**	87	48	34	0	1	35	1
**E**	37	0	0	1	18	0	0	0	9	2
**F**	90	48	47	27	31	15	0	25	87	78
**G**	8	9	3	1	45	1	43	0	18	5
**H**	33	25	24	12	100	8	0	5	149	31
**I**	31	**49**	40	0	22	19	10	22	54	39
**K**	7	20	4	0	4	0	0	0	7	4
**L**	46	73	44	0	97	52	45	52	**106**	**53**
**M**	150	37	26	30	50	16	0	16	40	38
**N**	56	10	32	19	38	22	21	30	24	1
**P**	6	10	0	0	13	0	0	0	143	29
**Q**	19	26	83	235	20	0	0	0	20	17
**R**	74	58	48	78	94	18	53	**382**	93	139
**S**	4	6	30	129	22	1	**121**	39	10	2
**T**	4	1	6	18	2	0	5	2	25	0
**V**	29	47	30	0	170	**244**	80	0	40	58
**W**	213	122	140	165	7	96	40	60	35	31
**Y**	**60**	17	16	42	**209**	95	0	0	18	316

VRC 19-29 — Score E1L$_{396-405}$ MYTYFSNTIL= 699

	P1	P2	P3	P4	P5	P6	P7	P8	P9	P10
**A**	1	17	0	0	6	2	0	0	0	23
**C**	36	22	1	0	19	11	0	0	50	4
**D**	8	19	0	0	42	19	0	0	8	29
**E**	0	20	0	4	0	3	0	15	5	3
**F**	12	18	4	2	**18**	6	0	11	27	3
**G**	10	23	0	0	10	11	1	0	45	12
**H**	27	2	5	0	6	1	0	0	36	15
**I**	82	12	5	1	23	0	4	19	**210**	40
**K**	1	16	0	0	1	2	0	0	0	16
**L**	67	2	5	0	14	0	3	3	36	**21**
**M**	**64**	8	3	1	5	1	0	0	33	19
**N**	21	5	0	0	12	31	**64**	10	18	8
**P**	6	0	0	0	3	0	0	0	14	1
**Q**	5	14	0	0	9	0	0	0	41	12
**R**	11	4	14	0	27	0	9	8	28	10
**S**	9	0	8	0	21	**55**	1	7	8	2
**T**	8	3	**161**	44	12	6	2	**31**	7	0
**V**	79	9	8	2	2	2	3	11	83	18
**W**	8	2	2	8	113	22	3	0	13	8
**Y**	16	**36**	14	**39**	62	8	0	0	8	20

VRC 19-36 — Score A6L$_{75-84}$ SFWFLKSGAV= 948

	P1	P2	P3	P4	P5	P6	P7	P8	P9	P10
**A**	0	14	0	9	0	3	30	54	**312**	0
**C**	49	10	0	0	12	3	0	0	10	0
**D**	29	0	10	0	20	26	0	0	7	1
**E**	66	3	0	0	0	11	0	0	13	0
**F**	11	**15**	0	**92**	29	0	0	6	0	8
**G**	0	4	0	0	4	37	24	**166**	0	27
**H**	0	2	0	0	15	10	0	1	96	58
**I**	25	0	0	15	155	0	16	14	17	90
**K**	33	19	3	71	75	**197**	19	0	1	8
**L**	55	9	10	18	**11**	48	21	1	29	89
**M**	66	0	0	0	5	2	2	0	5	2
**N**	8	9	0	13	26	5	20	6	1	99
**P**	2	49	0	77	168	83	24	2	122	2
**Q**	0	18	0	58	0	49	0	2	13	15
**R**	107	94	71	117	146	138	14	96	29	101
**S**	**1**	11	8	1	28	0	**19**	46	23	0
**T**	1	0	0	0	0	0	44	31	12	0
**V**	1	2	0	0	7	6	2	0	4	**0**
**W**	43	113	**135**	23	137	85	13	8	2	27
**Y**	0	9	0	7	8	109	0	7	0	183

VRC 47-38 — Score F13L$_{361-370}$ DWVSSHSKSL= 2593

	P1	P2	P3	P4	P5	P6	P7	P8	P9	P10
**A**	33	103	44	21	125	14	134	24	21	21
**C**	33	0	34	88	57	20	38	10	72	49
**D**	**88**	196	14	20	31	22	1	8	8	60
**E**	18	12	14	5	28	13	3	9	10	19
**F**	84	103	59	274	35	75	48	65	166	91
**G**	30	157	25	36	64	31	30	13	19	26
**H**	124	242	14	17	236	**370**	58	24	81	120
**I**	93	76	27	141	243	186	141	48	69	142
**K**	15	59	42	357	24	67	46	**383**	40	15
**L**	38	124	74	82	37	114	34	118	88	**53**
**M**	144	70	46	31	318	45	107	58	37	92
**N**	70	232	39	307	10	217	295	4	3	134
**P**	365	324	139	26	155	306	134	156	71	9
**Q**	66	94	78	61	44	35	16	11	21	35
**R**	84	65	118	376	171	134	107	113	104	75
**S**	79	26	17	**331**	**329**	63	**352**	57	**23**	31
**T**	111	37	147	22	77	159	61	15	17	48
**V**	72	72	**371**	121	228	26	15	13	41	64
**W**	276	**293**	237	345	123	312	53	0	178	64
**Y**	209	57	15	121	48	128	15	29	87	347

Amino acid corresponding to identified peptide
