# Supplementary material for: GM-CSF Production Allows the Identification of Immunoprevalent Antigens Recognized by Human CD4+ T Cells Following Smallpox Vaccination
Source: PLoS One. 2011 Sep 9;6(9):e24091. doi: 10.1371/journal.pone.0024091 (PMC3170313; doi:10.1371/journal.pone.0024091)
Supplement: Table S2 — Peptides identified from positional scanning based biometrical analysis for each T cell clone. (PDF) [file pone.0024091.s003.pdf]

Table 2S. Peptides identified from positional scanning based biometrical analysis for each T cell clone

Clone	Peptide #	Sequence 1	2	3	4	5	6	7	8	9	10	Vaccinia gene	Score	Relat. Max Score	Activity GM-CSF	TNF-α
VRC19-16[a]	**TPI 1713-1**	**Y**	**I**	**D**	**A**	**Y**	**V**	**S**	**R**	**L**	**L**	**VACWR118**	**1526**	**0.71**	**++**	**++**
	2	F	T	V	R	L	V	S	R	N	Y	VACWR089	1383	0.64	-	-
	3	D	D	D	A	V	H	S	R	V	Y	VACWR170	1376	0.64	-	-
	4	L	R	D	L	V	Y	L	R	L	Y	VACWR080	1309	0.61	-	-
	5	A	V	R	A	F	L	L	R	H	Y	VACWR188	1295	0.60	-	-
	6	H	V	I	D	V	D	V	R	L	Y	VACWR201	1295	0.60	-	-
	7	F	F	Q	Q	K	D	K	R	F	Y	VACWR049	1279	0.60	-	-
	8	V	V	D	I	I	V	N	R	L	Y	VACWR098	1258	0.59	-	-
	9	Y	N	I	A	D	A	A	R	H	Y	VACWR129	1253	0.58	-	-
	10	D	V	L	I	V	V	S	R	H	L	VACWR138	1222	0.57	-	-
	11	I	D	V	Q	T	G	R	R	P	Y	VACWR150	1218	0.57	-	-
	12	F	R	F	E	N	V	K	R	D	Y	VACWR024	1211	0.56	-	-
	13	M	R	R	I	A	V	V	R	F	R	VACWR110	1211	0.56	-	-
	14	F	W	V	S	D	D	E	R	C	Y	VACWR052	1204	0.56	-	-
	15	E	V	D	A	L	R	S	R	I	R	VACWR148	1197	0.56	-	-
	16	Y	S	H	Q	L	V	S	S	I	Y	VACWR180	1196	0.56	-	-
	17	C	I	V	Q	S	V	L	R	D	I	VACWR085	1195	0.56	-	-
	18	E	I	L	R	N	Y	L	R	L	Y	VACWR051	1190	0.55	-	-
	19	L	L	S	Q	Y	L	S	R	V	S	VACWR144	1190	0.55	-	-
	20	W	C	Y	S	Q	V	N	K	R	Y	VACWR184	1190	0.55	-	-
VRC19-29	**TPI 1751-18**	**M**	**Y**	**T**	**Y**	**F**	**S**	**N**	**T**	**I**	**L**	**VACWR057**	**699**	**0.84**	**++**	**++**
	19	M	Y	T	I	D	S	S	T	I	Q	VACWR189	613	0.73	-	-
	20	I	Y	T	Y	D	R	V	D	I	Y	VACWR129	593	0.71	-	-
	21	E	Y	T	A	C	N	N	T	I	I	VACWR162	592	0.71	-	-
	22	M	E	T	D	R	E	N	A	I	I	VACWR079	589	0.70	-	-
	23	L	Y	T	T	I	E	Q	N	I	D	VACWR102	583	0.70	-	-
	24	V	L	T	T	D	G	F	Y	I	D	VACWR126	578	0.69	-	-
	25	I	D	T	T	V	T	I	N	I	I	VACWR201	578	0.69	-	-
	26	V	E	T	V	Y	W	S	R	I	C	VACWR126	569	0.68	-	-
	27	I	L	T	F	V	K	N	K	I	I	VACWR045	565	0.68	-	-
	28	V	N	T	V	Y	C	K	N	I	L	VACWR042	561	0.67	-	-
	29	N	Q	T	T	P	Y	N	F	I	A	VACWR165	559	0.67	-	-
	30	M	F	T	L	L	S	H	T	I	C	VACWR040	557	0.67	-	-
	31	L	V	T	S	F	T	N	D	I	L	VACWR160	556	0.66	-	-
	32	L	M	T	D	D	N	I	N	I	L	VACWR039	554	0.66	-	-
	33	L	E	T	Y	T	R	P	E	I	D	VACWR051	553	0.66	-	-
	34	V	L	T	G	Y	G	L	E	I	N	VACWR031	551	0.66	-	-
	35	Y	Y	T	T	Y	I	D	H	I	V	VACWR058	547	0.65	-	-
VRC19-36	TPI 1751-36	M	R	M	R	F	K	K	G	A	V	VACWR184	1000	0.65	-	-
	**37**	**S**	**F**	**W**	**F**	**L**	**K**	**S**	**G**	**A**	**V**	**VACWR125**	**948**	**0.61**	**++**	**++**
	38	R	E	R	G	I	K	Y	L	A	I	VACWR194	936	0.61	-	-
	39	R	E	I	K	I	Y	E	G	A	K	VACWR036	931	0.60	-	-
	40	N	K	F	S	F	K	R	G	A	Y	VACWR069	929	0.60	-	-
	41	I	E	I	I	P	K	I	K	A	Y	VACWR102	919	0.60	-	-
	42	L	I	T	P	R	Y	S	G	A	S	VACWR026	884	0.57	-	-
	43	L	E	R	Q	P	R	R	S	P	Y	VACWR142	858	0.56	-	-
	44	E	R	W	F	H	G	D	A	A	W	VACWR042	832	0.54	-	-
	45	K	R	W	R	R	F	D	G	P	C	VACWR163	813	0.53	-	-
	46	M	W	D	L	K	K	K	G	L	I	VACWR078	783	0.51	-	-
	47	R	R	W	L	K	I	K	R	D	Y	VACWR176	734	0.48	-	-
	48	G	Y	R	K	Q	K	M	G	P	I	VACWR025	728	0.47	-	-
	49	R	R	F	R	R	P	M	S	L	R	VACWR068	725	0.47	-	-
	50	R	M	R	F	K	K	G	A	V	L	VACWR184	713	0.46	-	-
VRC47-38	**TPI 1751-51**	**D**	**W**	**V**	**S**	**S**	**H**	**S**	**K**	**S**	**L**	**VACWR052**	**2593**	**0.76**	**++**	**++**
	54	T	I	V	K	S	L	N	K	I	Y	VACWR048	2451	0.72	-	-
	59	P	M	P	R	H	H	S	I	V	Y	VACWR026	2343	0.69	-	-
	60	A	L	W	S	A	H	N	K	L	Y	VACWR180	2332	0.69	-	-
	61	P	W	I	K	T	I	S	K	R	M	VACWR143	2237	0.66	-	-
	71	S	E	V	F	I	H	N	K	I	N	VACWR077	2229	0.66	-	-
	65	N	N	V	Q	S	L	I	K	F	I	VACWR125	2009	0.59	-	-
	66	M	P	V	K	G	K	R	K	D	I	VACWR155	1966	0.58	-	-
	68	H	N	V	S	S	L	V	K	N	V	VACWR057	1965	0.58	-	-
	63	V	I	V	R	N	L	N	K	I	I	VACWR073	1907	0.56	-	-
	69	S	H	V	K	S	V	T	K	S	S	VACWR117	1902	0.56	-	-
	64	Y	I	V	H	S	Y	L	K	N	Y	VACWR203	1897	0.56	-	-
	62	L	L	V	S	S	S	V	K	H	I	VACWR110	1876	0.55	-	-
	67	D	G	V	S	S	L	L	K	E	L	VACWR138	1869	0.55	-	-
	56	L	Y	V	K	A	L	T	K	N	Y	VACWR062	1854	0.55	-	-
	70	N	F	V	S	S	F	L	K	A	N	VACWR120	1851	0.55	-	-
	52	V	I	V	S	T	F	N	K	T	I	VACWR102	1838	0.54	-	-
	55	Y	I	V	V	N	P	M	K	M	I	VACWR122	1761	0.52	-	-
	53	N	I	V	K	D	L	K	K	I	I	VACWR103	1658	0.48	-	-
	58	D	R	V	L	N	I	N	K	N	G	VACWR186	1510	0.44	-	-
	57	L	L	V	N	N	H	L	G	I	G	VACWR010	1363	0.40	-	-

[a] 20 of 35 synthesized peptides are shown
++ Active at 10 and 1ug/ml
- No response
BA score: biometrical analysis score
VRC19-36, scores from Screen 1
